# Post-weaning shifts in microbiome composition and metabolism revealed by over 25 000 pig gut metagenome-assembled genomes

**DOI:** 10.1099/mgen.0.000501

**Published:** 2021-08-09

**Authors:** Daniela Gaio, Matthew Z. DeMaere, Kay Anantanawat, Toni A. Chapman, Steven P. Djordjevic, Aaron E. Darling

**Affiliations:** ^1^​ iThree Institute, University of Technology Sydney, Sydney, New South Wales, Australia; ^2^​ NSW Department of Primary Industries, Elizabeth Macarthur Agricultural Institute, Menangle, New South Wales, Australia

**Keywords:** carbohydrate active enzymes, co-assembly, gut microbiome, post-weaning, shotgun metagenomics, time series

## Abstract

Using a previously described metagenomics dataset of 27 billion reads, we reconstructed over 50 000 metagenome-assembled genomes (MAGs) of organisms resident in the porcine gut, 46.5 % of which were classified as >70 % complete with a <10 % contamination rate, and 24.4 % were nearly complete genomes. Here, we describe the generation and analysis of those MAGs using time-series samples. The gut microbial communities of piglets appear to follow a highly structured developmental programme in the weeks following weaning, and this development is robust to treatments including an intramuscular antibiotic treatment and two probiotic treatments. The high resolution we obtained allowed us to identify specific taxonomic ‘signatures’ that characterize the gut microbial development immediately after weaning. Additionally, we characterized the carbohydrate repertoire of the organisms resident in the porcine gut. We tracked the abundance shifts of 294 carbohydrate active enzymes, and identified the species and higher-level taxonomic groups carrying each of these enzymes in their MAGs. This knowledge can contribute to the design of probiotics and prebiotic interventions as a means to modify the piglet gut microbiome.

## Data Summary

The data were deposited in the National Center for Biotechnology Information Sequence Read Archive under project accession number PRJNA526405. Workflows and code used for the preliminary data processing are available in the GitHub repository (https://github.com/koadman/metapigs). Code used for the data analysis and visualization are available in the GitHub repository (https://github.com/GaioTransposon/metapigs_dry).

**Impact Statement**

The aim of this work was to assemble a large amount of metagenomic data into metagenome-assembled genomes (MAGs), and to use the MAGs to describe the developing gut microbial composition of post-weaning piglets, from a genomic and a functional perspective. A large number of shared species were found to undergo the same shifts in distinct hosts during the first 5 weeks after weaning. This type of fine, species-level tuning of the gut in piglets was not measured before and can be used to choose (or avoid) a time window for treatment with specific probiotics. We also confirmed confounding factors (e.g. breed, small differences in age) to significantly correlate with microbial composition, and we report the duration of their detection. This can guide researchers to make the proper adjustments to their animal trials, so as to minimize noise in the signal. Ultimately, we obtained a description of the carbohydrate enzyme repertoire of these post-weaning piglets, by tracking temporal abundance shifts of nearly 300 carbohydrate active enzymes and their mapping, hence, specificity, to taxonomic groups, knowledge which can be applied to manufacture feed with the aim of tweaking the abundance of specific (e.g. health-promoting, low carbon emission) taxa.

## Introduction

Advances in sequencing technology and computational analysis enable the study of whole microbial communities from a given environment in a high-throughput manner, bypassing the need to cultivate individual organisms. High-throughput microbial community profiling is generally approached in a targeted (e.g. 16S rRNA) or untargeted (shotgun) manner.

Only a tiny fraction of known bacterial species are represented in genome sequence databases and, in particular, difficult-to-culture and non-pathogenic organisms remain greatly underrepresented [1–3]. On the contrary, taxa of interest for their pathogenicity or potential in the biotech sector are more often sequenced and, therefore, tend to be better represented in public databases. Although an increasing diversity of reference genomes is becoming available, reference-based methods are not ideal for the discovery of new genomes, as they depend, directly or indirectly, on existing databases [4]. Moreover, proof of the nature of functional redundancy in the microbiome [5, 6], the early documented within-species genomic diversity [7], coupled with its striking functional implications [6, 8, 9], should make us ever more aware that important compositional and functional features may be masked completely when the view of the microbiome is restricted to a small conserved gene fragment and the assumptions on its conservation, as in the 16S rRNA amplicon profiling technique.

Shotgun metagenomic sequencing, however, allows a much more complete view of the microbial community. The technique uses assembly and binning for the reconstruction of genomes from metagenomic reads, but it comes with many technical challenges. Among the main challenges are the detection of less prevalent taxa within the sample [10], and the high sequence similarity among strains of the same species, making assembly a particularly challenging task [11, 12]. However, various techniques have been developed to overcome these obstacles. Previous work in computational metagenomics has established that repeated sampling from an environment or subject can facilitate the reconstruction of genomes from species [13, 14] and strains [15] in the microbial community. Two major steps are typically involved in the data analysis: assembly and binning. The joint assembly of samples from an individual host is called co-assembly, while binning consists in the grouping of co-assemblies into metagenome-assembled genomes (MAGs) in the process of differential coverage binning [16]. The abundance per sample is then inferred from coverage depth and/or *k*-mer frequencies [17]. The rationale behind this type of binning method is based on the observation that the abundance of genetic material from the same organism changes in one subject or environment over time in a highly correlated manner [13, 14, 16, 18]. There are two reasons that time-series binning facilitates the resolution of genomes: (i) strains are known to persist for long periods of time (i.e. decades) [19], and as such the abundance of the same organism in a subject or environment changing over time in a highly correlated manner improves the inference of MAGs; (ii) at least in humans, over half of all species in the gut are represented by single strains [20]; therefore, co-assembly of time points boosts the per-genome coverage.

The present work builds upon a large collection of post-weaning porcine gut samples for which we previously described the microbial community composition using an assembly-free phylogenetic profiling technique [21]. We reconstruct genomes from this large sample collection and analyse how those genomes change in abundance across time and treatment cohorts. To do so, we created co-assemblies for each individual host, pooling all the time point samples available from each subject, thereby increasing the power to reconstruct genomes from low-abundance microbes. In this study, we describe the associations we found between specific genomes and the post-weaning aging process in piglets, as well as the carbohydrate metabolism during the post-weaning period.

## Methods

### Metagenomic samples

Below, we briefly summarize the origin of the samples, but we refer to our previous report [21] for a thorough description of the animal trial and the sample processing workflow. Subjects were post-weaning piglets (*n*=126) from which faecal samples were collected between 1 and 10 times during a 40 day period. Piglets were aged 22.5±2.5 days at the start of the trial. The piglets were distributed over six treatment cohorts: a placebo group (control *n*=29); two probiotic groups (D-Scour *n*=18; ColiGuard *n*=18); one antibiotic group (neomycin *n*=24); and two antibiotic-then-probiotic treatment groups (neomycin+D-Scour *n*=18; neomycin+ColiGuard *n*=18). Additionally, 42 faecal samples derived from the piglets’ mothers, 18 samples derived from three distinct positive controls and 20 negative controls were included. All piglets were sampled weekly, while a subset of the piglets (8 per cohort; *n*=48) was sampled twice weekly. In total, mothers were sampled once, while piglets were sampled between 1 and 10 times (median=6.0; mean=6.11). As previously described [21], samples were homogenized immediately after collection and stored at −80 °C until thawed for further processing. Extraction of DNA was performed with the PowerMicrobiome DNA/RNA EP kit (Qiagen) and libraries were prepared from genomic DNA using the Hackflex method [22]. Libraries were normalized, pooled and sequenced on three Illumina NovaSeq S4 flow cells. The data were deposited in the National Center for Biotechnology Information Sequence Read Archive under project accession number PRJNA526405.

### Sequence data processing

A total of 911 samples were sequenced, generating a mean of 7 581 656 (median=3 936 038) paired-end reads per sample for a total of 27.2 billion paired-end reads. BBDuk [23] (v38.22) was used for adapter trimming (parameters: k=23 hdist=1 tpe tbo mink=11), PhiX DNA removal (parameters: k=31 hdist=1) and quality filtering (parameters: ftm=0 qtrim=r trimq=20). Quality assessment was performed with fastqc [24] (v0.11.18) and multiqc [25] (v1.8).

### Pooled assembly and binning

Adapter-trimmed and quality-filtered paired-end reads from all samples were grouped by subject and assembled using megahit [26] (v1.1.3) (parameters: --min-contig-len=5 --prune-level=3 --max-tip-len=280), yielding a combined total of 8 389 418 contigs. Reads were mapped back to assembly contigs using bwa-mem [27] (v0.7.17-r1188). Format conversion to bam format was performed with SAMtools [28] (v1.9). Metagenome binning of the co-assemblies was performed for each subject (i.e. each pig, positive control and all negative controls were separately co-assembled) with MetaBAT2 [29] (v2.12.1) (parameters: --minContig 2500).

### MAG quality assignment

Quality analysis of MAGs was performed with CheckM [30] (v1.0.13) following the lineage-specific workflow (lineage_set).

### MAG taxonomic assignment

Taxonomic clustering of MAGs was performed with Kraken2 [31, 32] (v2.0.8) using, in parallel: a prebuilt 8 Gb database (MiniKraken DB_8 GB) constructed from bacterial, viral and archaeal genomes from RefSeq version Oct. 18 2017, and the Genome Taxonomy Database (GTDB) [33] (release 89.0). All MAGs were assigned a taxon at the species level. Higher taxonomic levels of the GTDB were retrieved from the latest release (release 89.0) of the bacterial and archaeal database publicly available at the GTDB website (https://gtdb.ecogenomic.org/). Phyla composition profiles were obtained from presence/absence counts of MAGs, ignoring their relative abundances in each sample, using MAGs that were taxonomically assigned with either GTDB or CheckM. We refer to these as GTDB clustered MAGs and CheckM clustered MAGs.

### MAG dereplication

Because MAGs were constructed independently for each host, highly similar MAGs are expected to be present in the data, representing the same species or strain recovered from different hosts. To group the MAGs into collections that represent MAGs of the same species or strain we carried out bin dereplication using dRep (v2.3.2) [34]. All MAGs derived from pig samples were dereplicated with dRep [34] (parameters: --checkM_method=lineage_wf --length=500 000). All other parameters were set to default. The clusters of similar bins produced by dRep at 95 % identity (primary clusters) and at 99 % identity (secondary clusters) were used for further analysis as described below. We refer to these as 95 % average nucleotide identity (ANI) MAG clusters and 99 % ANI MAG clusters.

### Data analysis

Analyses of MAGs were performed for the following sets of MAGs: (i) nearly complete genomes (≥90 % completeness and ≤5 % contamination) as estimated and taxonomically categorized by CheckM analysis (*n*=12.4×10^3^); (ii) length filtered and ANI clustered MAGs from dRep analysis (*n*=22.4×10^3^); (iii) all (unfiltered) MAGs taxonomically categorized by GTDB (*n*=51.2×10^3^). Non-metric multidimensional scaling (NMDS) and network analysis were performed with PhyloSeq [35] using the median sequencing depth to normalize samples. Bray–Curtis value was used as a distance measure. Separately from the ordination analysis performed with PhyloSeq, we ran principal component analysis (PCA) with data normalized by proportions and transformed with the centred log-ratio. In the latter case, the data underwent the following transformations: (i) library size normalization by proportions; (ii) sum of counts from MAGs falling under the same MAG (taxonomic or ANI) group assignment; (iii) mean by sampling time point and cohort; (iv) centred log-ratio transformation.

Microbial diversity assessment and microbial abundance heatmaps were generated using GTDB clustered MAGs. After the exclusion of samples with low read counts (approximately <10 000) (*prune_samples* function), samples were rarefied (*rarefy_even_depth* function) (PhyloSeq v1.28.0). To compare microbial diversity scores of samples from different time points, *t*-tests were applied, and significance values were adjusted with the Bonferroni method. To generate the heatmaps, taxa were filtered based on abundance, prevalence and within-samples variation, as indicated in the figure legends.

Age, breed, co-housing and piglet weight were tested for correlation with microbiome composition. Samples were pruned and rarefied as described above and PCA was performed with PhyloSeq (v1.28.0). As breed and age were confounded factors, we additionally performed PCA on subsets of equal age or breed. The resulting eigenvectors were tested to assess the significance of the correlations. For the categorical variables age, breed and co-housing, Dunn tests were performed. For the continuous variable weight, the Spearman’s rank test was performed. Significance values were adjusted by Bonferroni correction for multiple testing.

### Differential abundance analysis

siamcat [36] was employed to determine differentially abundant GTDB clustered MAGs between groups. The data were normalized by proportions prior to analysis with siamcat. Unsupervised abundance and prevalence filtering is run prior to the association testing. The abundance was normalized, and all GTDB clustered MAGs underwent analysis with siamcat. Associations of species with groups were found by running the siamcat *check.associations* function, which finds associations of single species with groups using the nonparametric Wilcoxon test and reporting associations and their strength using significance, prevalence shift and generalized fold change (fc) metrics, the latter calculated as the geometric means of the differences between quantiles [37]. The Benjamini–Hochberg method was used to control the false discovery rate (i.e. the expected proportion of false discoveries amongst the rejected hypotheses).

### Gene function analysis

Protein prediction from MAGs was performed with Prodigal [38] (v2.6.3). The predicted proteins were clustered with cd-hit [39] (v4.6) using 90 and 100 % identity. Predicted proteins were mapped against a custom database of the UniRef90 database (release June 2020) using diamond [40] (v0.9.31). Before mapping, the database was filtered to contain only error-corrected sequences matching the uniref90_ec_filtered list from HUMAnN2 [41] (/utility_mapping) (v2.8.1). To run the filtering, SeqTK [42] (v1.3-r106) was used.

Additionally, predicted proteins were mapped against the carbohydrate active enzyme (CAZy) database [43] with dbCAN2 [44] (v2.0.11), which utilizes diamond [40] and hmmer [45]. The proportion of species per enzyme ID was derived as follows: MAGs were grouped by subject, enzyme ID and GTDB-assigned species; distinct groups were selected and a count was obtained; the sum of distinct species falling within one enzyme ID was obtained and the proportion was derived.

### Analysis workflow and scripts

In order to manage the processing of the data in the high performance computing environment (sequence data processing, pooled assembly, binning, MAGs quality assessment), Nextflow [46] was used. For the installation and management of environments, conda [47] was used (v4.7.12). R [48] and the following R packages were used for the data analysis and visualization: ape [49], circlize [50], cluster [51], cowplot [52], data.table [53], dplyr [54], EnvStats [55], factoextra [56], forcats [57], ggbiplot [58], ggplot2 [58], ggpubr [59], ggrepel [60], gplots [61], gridExtra [62], magrittr [63], matrixStats [64], microbiome [65], openxlsx [66], pheatmap [67], phyloseq [35], plyr [68], purr [69], RColorBrewer [70], readr [71], readxl [72], reshape [73], robCompositions [74], scales [75], seqinr [76], siamcat [36], splitstackshape [77], stringr [78], tidyr [79] and tidyverse [80].

A schematic workflow of the data processing and analysis is represented in Fig. S1 (available with the online version of this article). Workflows and scripts used for the data processing are available in the GitHub repository (https://github.com/koadman/metapigs). Scripts used for the data analysis and visualization are available in the GitHub repository (https://github.com/GaioTransposon/metapigs_dry).

## Results

A total of 51 170 MAGs from 911 samples were generated in this study, 92.96 % (*n*=47 569) of which derived from samples of post-weaning piglets, 5.24 % (*n*=2680) from samples of the piglets’ mothers, and 1.81 % (*n*=926) from negative and positive control samples. Distribution of bins over subjects and cohorts are displayed in Fig. S2.

### Completeness and contamination of MAGs

**Fig. 1.**
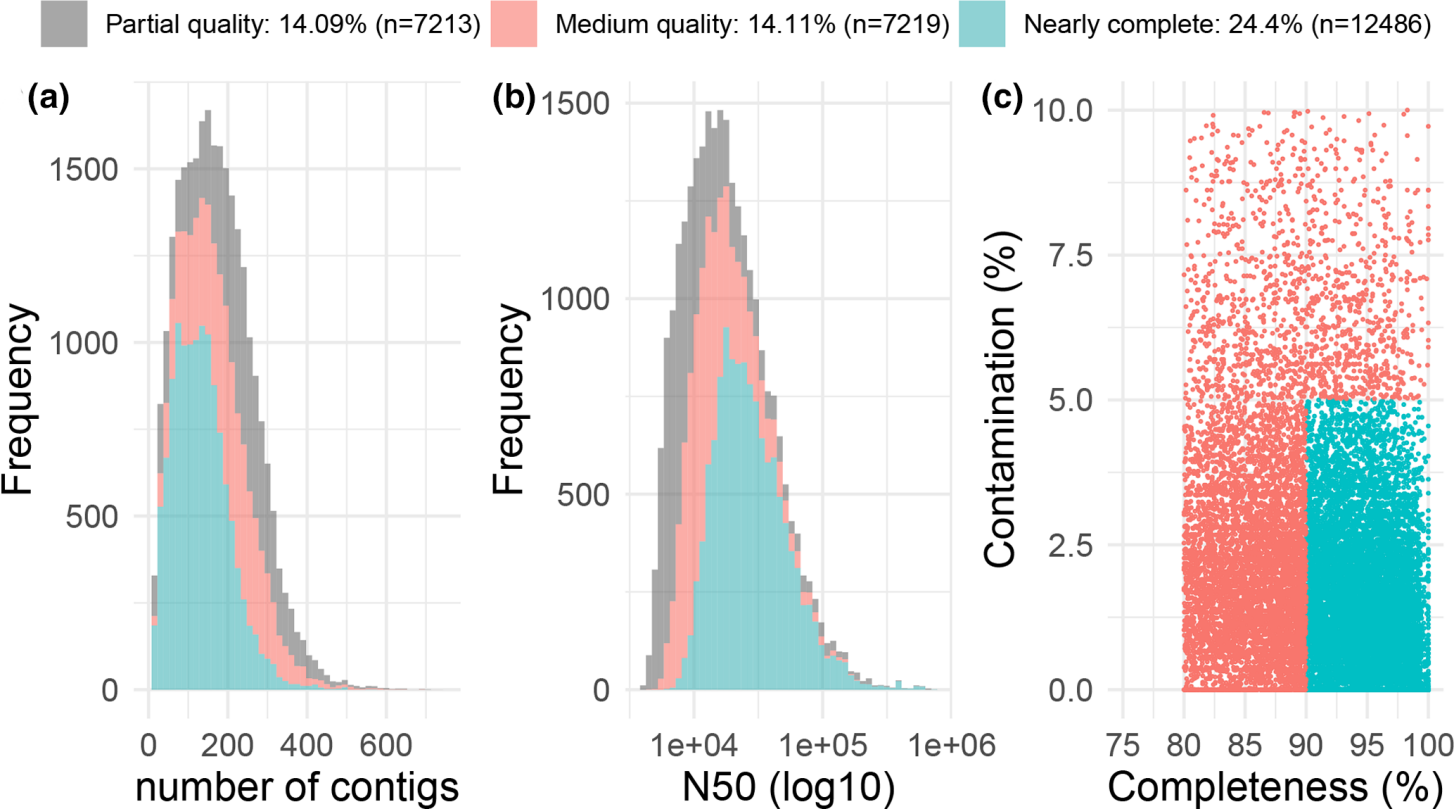
Quality of MAGs. Distribution of contig counts (**a**), contig N50 lengths (**b**), and completeness and contamination rates over MAGs of partial quality (grey) and medium quality (pink), and nearly complete MAGs (blue) (**c**). Partial and medium quality MAGs have a level of completeness between 60 and 80 %, and between 80 and 90 %, respectively, and a rate of contamination of <10 % and ≤10 %, respectively. MAGs with a ≥90 % completeness and a ≤5 % contamination are considered nearly complete as by the Genomic Standards Consortium MIMAG standards [84].

According to quality analysis with CheckM [30], 46.5 % (*n*=23 798) of the total MAGs (*n*=51 170) were classified as ≥70 % complete with a ≤10 % contamination rate, while 24.4 % (*n*=12 486) were classified as nearly complete genomes as by the Bowers *et al*. [10] definition of MAGs with ≥90 % completeness and ≤5 % contamination (Fig. 1). Three hundred and thirty MAGs were ≥99 % complete and had a ≤0.1 % contamination rate. The MAGs with ≥60 % completeness and ≤10 % contamination rate ranged in size from 442 kb to 6.4 Mb (median=1.9 Mb), and their N50 values ranged from 4 to 728 kb (median=16 kb).

### MAG ANI clusters and taxonomic clusters

As a reference-free technique of MAG generation was adopted, we grouped the MAGs into clusters of similar genomes using two methods: (i) 95 and 99 % sequence similarity using the genome dereplication tool dRep [34]; and (ii) taxonomic clustering with Kraken2 [32] against the GTDB [33, 81]. Using the first method, nearly half of the MAGs (43.8 %) passed the established length-filtering threshold (500 000 nt) and were assigned a cluster ID based on ANI. A total of 1267 unique primary clusters (95 % ANI) and 4480 unique secondary clusters (99 % ANI) were obtained. Primary or secondary clusters that were found in only one subject were defined as unique, whereas clusters that were found in more than one subject were defined as common. Primary clusters (95 % ANI) were common among subjects at a higher rate than secondary clusters (99 % ANI): 98.6 and 86.2 % among piglets, and 91.4 and 72.1 % among the mothers, respectively, delineating strain specificity within hosts (Fig. S3).

The second method applies clustering via taxonomic assignment using the most comprehensive bacterial and archaeal taxonomic database that is currently available. Based on GTDB clustering of MAGs derived from positive control samples (three positive control types; eight replicates within each), MAG taxonomic assignments matched the expected profile and the profile obtained from analysis of the reads with MetaPhlAn2 [82] reported in our previous study [21] (Fig. S4).

### High-level taxonomy of the piglet gut microbiome

According to taxonomic assignment of the nearly complete genomes with CheckM [30], the average post-weaning piglet gut microbiome is composed of the following phyla in the following proportions: *Firmicutes* (63.6 %), *Bacteroidetes* (13.2 %), *Tenericutes* (9.2 %), *Actinobacteria* (6.8 %), *Proteobacteria* (2.2 %), *Euryarchaeota* (2.1 %), *Spirochaetes* (1.8 %), *Chlamydiae* (0.8 %) and *Synergistetes* (0.5 %). CheckM taxonomic clustering resolved 91.28 % of the nearly complete MAGs to the order level, the most common taxonomic orders above 1 % being: *Clostridiales* (56.4 %), *Bacteroidales* (12.9 %), *Erysipelotrichales* (8.2 %), *Coriobacteriales* (5.4 %), *Lactobacillales* (5.4 %), *Selenomodales* (3.4 %) and *Methanobacteriales* (1.8 %). According to taxonomic assessment of MAGs against the GTDB, the genus *Prevotella* took up the largest proportion, with 10.1 % of the total genera composition. The most common phyla, genera and species of the piglet microbiome are shown in Fig. S5.

### Time trend

The predominant variation we observed in the piglet microbiomes is associated with the aging of the piglets. Both NMDS analysis and network analysis of samples with PhyloSeq [35] showed samples clustering by collection time point (Fig. S6). Samples were the most scattered in PCA at the start of the trial (*t*0 variance=0.1669, *n*=122) when piglets were aged between 3 and 4 weeks old, and samples clustered more closely at later time points (*t*2 variance=0.0625, *n*=115; *t*4 variance=0.0405, *n*=105), shifting most prominently along the first NMDS axis (*t*-test; Bonferroni adjusted significance: *t*0*–t*2 *P* <0.0001; *t*2*–t*4 *P* <0.0001) (Fig. S6). The temporal shift was evident in the results from all of the various approaches we applied to filter and cluster MAGs into groups; hence, the final number of MAGs included in the analysis (CheckM 12.4×10^3^; dRep 22.4×10^3^; GTDB 51.2×10^3^) (Fig. S7).

### Remarkable tightly regulated compositional shift

**Fig. 2.**
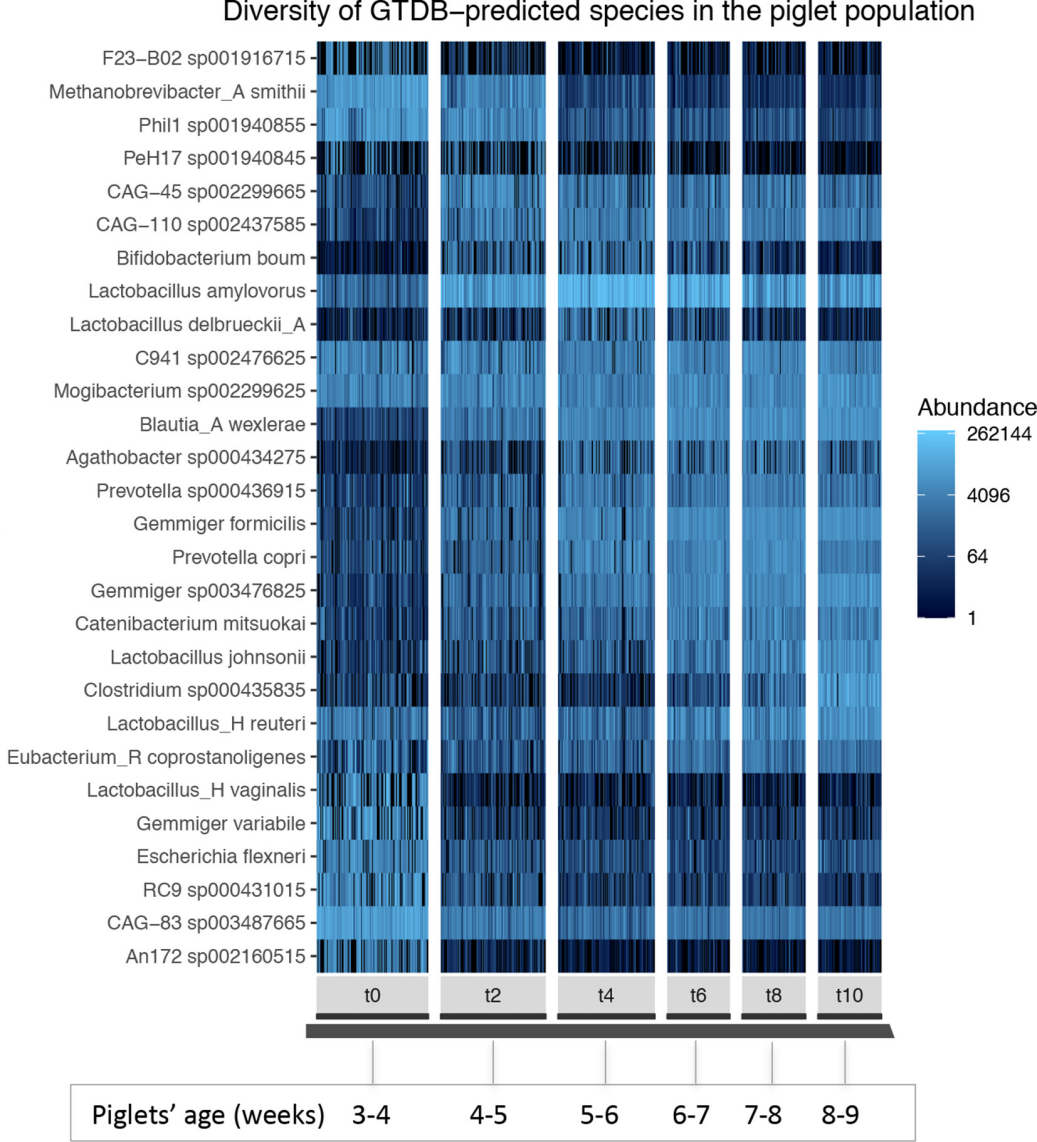
Microbial species abundance profile of the piglet gut over time. Panels represent time points from *t*0 (piglets aged 3 to 4 weeks old) to *t*10 (piglets aged 8 to 9 weeks old). Samples were pruned (exclusion of samples with approximately <10 000 read counts) and normalized by rarefaction. Prior to plotting, taxa were filtered to include the most abundant taxa present in at least 20 % of the piglet samples. Of these, the 35 taxa with the highest variance among samples were plotted. Analysis was performed with PhyloSeq.

We found the aging-associated compositional shift throughout the length of the study to be consistent among hosts, independent of treatment, and marked by changes that were clearly associated with particular taxonomic groups (Figs 2 and S8).

**Fig. 3.**
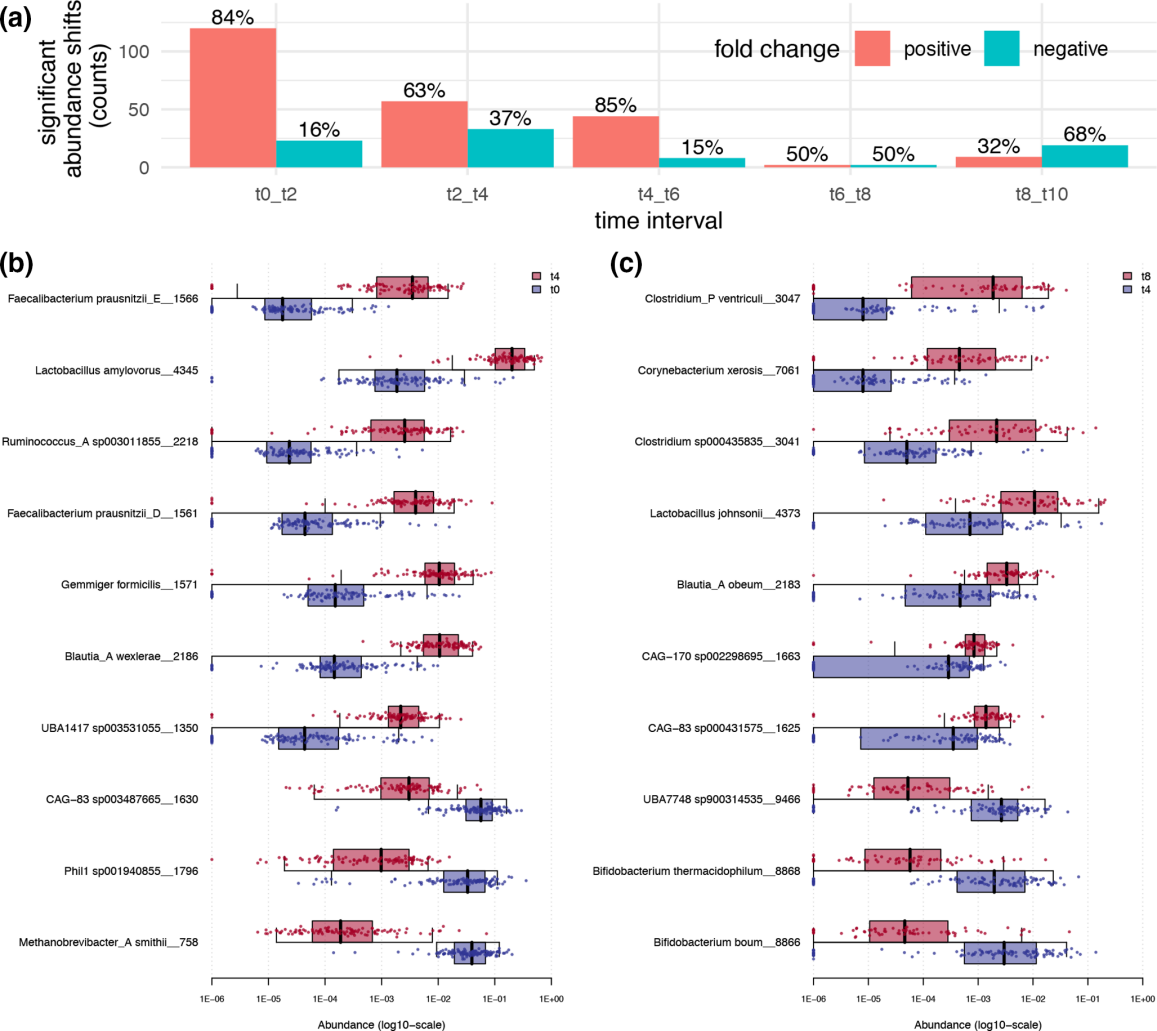
Significantly changing GTDB species with time. Significant shifts (alpha=0.05) were determined from comparison of GTDB clustered MAGs abundance in samples from distinct time points with siamcat. The Benjamini–Hochberg method was used to control the false discovery rate. (**a**) Percentages of negative fc and positive fc shifts are reported on top of the bar plots for each time interval, where two consecutive time points were compared. (**b**) Shown are the top 10 significantly changing GTDB taxonomically clustered MAGs at the species level, between time points 0 and 4 (2 week interval); and (**c**) between time points 4 and 8 (2 week interval). Points show normalized and log-transformed abundance within each subject and GTDB-predicted species.

Differential abundance between time points and statistical analysis of the changes over time was obtained with siamcat [36]. Time points analysed were at consecutive weeks from the start (*t*0): *t*0, *t*2, *t*4, *t*6, *t*8 and *t*10. The top 10 significantly shifting species in piglets between *t*0 and *t*4, and between *t*4 and *t*8, are shown in Fig. 3. Correlations with *P* <0.05 after false discovery rate correction (Benjamini–Hochberg method) were considered significant. Significance values, generalized fc and other metrics are provided in Table S1.

A total of 143 species were found to significantly shift in abundance (*P* <0.001; 84 % positive fc) in the piglet population between *t*0 and *t*2, (piglets aged 3–4 weeks and 4–5 weeks, respectively) (Table S1), among which we found: *Blautia* _A *wexlerae* (fc=+1.4), CAG-83 sp003487665 (fc=−0.9), *Lactobacillus amylovorus* (fc=+1.3), CAG-45 sp002299665 (fc=+1.7), CAG-110 sp002437585 (fc=+1.7). Significant shifts in abundance were found for 90 species (*P* <0.001; 63 % positive fc) between *t*2 and *t*4 (Table S1), among which we found: *Methanobrevibacter* _A *smithii* (fc=−1.6), Phil1 sp001940855 (fc=−1.3), *Prevotella copri* (fc=+1.0), *Prevotella* sp000434515 (fc=+1.3), *Lactobacillus amylovorus* (fc=+0.7). Fifty-two species significantly shifted in abundance (*P* <0.001; 85 % positive fc) between *t*4 and *t*6 (Table S1), among which we found: *Corynebacterium xerosis* (fc=+1.4), *Blautia* _A *obeum* (fc=+1.1), *Blautia* _A sp000285855 (fc=+0.8), *Clostridium* sp000435835 (fc=+1.0), *Clostridium* _P *ventriculi* (fc=+1.4). The least significant shifts in abundance (*n*=4) were found between *t*6 and *t*8 (*P*<0.05; 50 % positive fc) (Table S1). These were: UBA7748 sp900314535 (fc=−0.9), *Clostridium* sp000435835 (fc=+0.8), *Prevotella copri* (fc=+0.3) and *Bifidobacterium boum* (fc=−0.8). The number of species significantly shifting in abundance (*P* <0.001; 32 % positive fc) was again higher (*n*=28) for the last time interval (*t*8*–t*10) when piglets were aged between 7–8 and 8–9 weeks. During this interval, the most significantly changing species were: *Clostridium* sp000435835 (fc=+1.4), *Corynebacterium xerosis* (fc=+1.2), *Prevotella copri* _A (fc=−0.7), *Prevotella copri* (fc=−0.5), *Prevotella* sp000434515 (fc=−0.5) (Table S1). Notably, of all *Prevotella* species (*n*=16) significantly increased in abundance during the first or the second week, nearly all (*n*=13) decreased during the last time interval (false discovery rate adjusted *P* <0.05) (Fig. S9). All significance values and confidence intervals are given in Table S1.

### Diversity

During the first week (*t*0–*t*2) (piglet age range: 3.5±0.5 to 4.5±0.5 weeks) species richness increased (Shannon ∆ _*t*2-*t*0_=0.53±0.12; Bonferroni adjusted *P* <0.00001) (Fig. S10, Table S1). Between *t*2 and *t*4 (piglets age range: 4.5±0.5 to 5.5±0.5 weeks) a loss of species evenness was recorded (Simpson ∆ _*t*4-*t*2_=−0.04±0.02; Bonferroni adjusted *P* <0.00001). The following week (*t*4–*t*6) (piglets age range: 5.5±0.5 to 6.5±0.5 weeks) an increment of species richness (Chao1 ∆ _*t*6-*t*4_=37.20±15.13; Bonferroni adjusted *P* <0.00001) was observed (Fig. S10; Table S1).

### Effects of age, breed, co-housing and weight

Age and breed were found to correlate with microbial composition (Fig. S11, Table S1). Piglets of the same breed (D*×*L) and two age groups (3 days difference between the two groups) separated by age at *t*0 in principal component 1 (PC1) (16.9 % variation explained; Dunn test; Bonferroni adjusted *P*=0.001) and at *t*2 in PC1 (14.5 % variation explained; Dunn test; Bonferroni adjusted *P*=0.002) and in PC2 (10.0 % variation explained; Dunn test; Bonferroni adjusted *P*=0.026) (Fig. S11, Table S1). These age groups were compared with siamcat [36] to discover taxa that exhibit an age association in their abundances. A list of taxa that were found to be mildly associated with age at each time point and the significance of these associations are reported in Table S1.

Breed was also found to significantly associate with microbiome composition when piglets of the same age and two breeds (D*×*L and D*×*LW) were compared. Significance was reached in PC2 (variation explained: 10.1 %) at *t*0 (Dunn test; Bonferroni adjusted *P*=0.001) and in PC1 (variation explained: 12.9 %) at *t*2 (Dunn test; Bonferroni adjusted *P*=0.024) (Fig. S11, Table S1). Piglets of the same age and two breed groups were compared with siamcat to find taxa for which abundances were associated with breed. Neither at 95 % confidence (alpha 0.05) nor 80 % confidence (alpha 0.20) was the association of microbial composition with breed found to be attributable to specific taxa (Table S1). Weak correlations were found between taxon abundances and weight (variance explained <4.3 %; Spearman’s rank *rho* range −0.349–0.246; *P* values range 0.003–0.043) (Table S1).

### Predicted proteome of the piglet

Gene prediction from all MAGs (*n*=51 170) yielded 70 696 284 predicted ORFs. The predicted protein sequences fell into 24.4 million clusters at 100 % amino acid identity and into 6.9 million clusters at 90 % amino acid identity. Homology searching of all the predicted proteins against the CAZy database with hmmer identified 2 049 008 predicted proteins that are potentially involved in carbohydrate metabolism (*E* value <1×10^−15^; coverage median=94.3, mean=87.0). Of these enzymes, 1 023 807 were glycoside hydrolases (GHs), 716 644 glycosyl transferases (GTs), 26 544 carbohydrate binding modules (CBMs), 253 525 carbohydrate esterases (CEs), 16 636 polysaccharide lyases (PLs), 8626 enzymes with auxiliary activity (AA), 3064 S-layer homology domain (SLH) enzymes and 162 were cohesins. We report the sequence identity against the CAZy database for each class of enzymes and the distribution of carbohydrate enzymes across phyla in Fig. S12.

### CAZy species-specificity and distribution across the pig population

Two hundred and ninety-four unique CAZy enzymes were found in our dataset. As each enzyme prediction was associated with a MAG and each MAG was separately classified against the GTDB, the two sources of information were joined as described in Methods. We display the prevalence of each enzyme in the pig population (*y*-axis), while on the *x*-axis, we report the highest proportion a distinct species was found for each enzyme ID (Fig. S13). Below, we describe each enzyme class in terms of prevalence in the pig population and its distribution among species.

Enzymes of the CE class (*n*=16) frequently occurred among subjects, with >100 subjects carrying this class of enzymes (median=166; mean=148). These enzymes tend to be species generic (median=5.5; mean=14.2), as they fall on the left side of the plot, showing a broad distribution across multiple species. Out of all genera, *Prevotella* carried the most of these enzymes (11.5 %).

Similarly, of the PL enzyme class (*n*=26), nearly half formed a cluster in the centre left side of the plot, indicating a high prevalence among subjects (median=93; mean=81) and a broad distribution across species (median=20.8; mean=32.2). PL enzymes were carried mostly by the genus *Prevotella* (40.6%).

Enzymes of the GT class (*n*=63) are prevalent among the pig population (median=121; mean=107) and have a broad distribution across species (median=16.0; mean=25.6). The majority of these enzymes (*n*=47) were found among species of the genus *Prevotella *, 27 of which were also found in *Gemmiger *. Species of these genera had the highest variety of enzymes, as well as the highest number of genes (>200) per genome (Fig. S14).

GH enzymes (*n*=126) were the most represented enzymes in our dataset and over 70 % of these enzymes fell on the top left of the plot, showing a high frequency across subjects (median=154; mean=131) and a high distribution across species (median=11.2; median=20.4). One hundred and thirteen of the 126 GH enzymes are found in the genus *Prevotella *, 96 of which were shared with the genus *Blautia* _A.

CBM enzymes (*n*=54) were moderately prevalent enzymes in the pig population (median=41; mean=65), and the most heterogeneous among the enzyme classes in terms of distribution across different species (median=27.0; mean=37.9); therefore, they appeared as scattered across the plot. Also, for this class of enzymes, the majority (*n*=30) were found among species of the genus *Prevotella *, and partially shared with *Gemmiger* (*n*=8). The genus CAG-269 carries three CBM enzymes neither *Prevotella* nor *Gemmiger* carry in their MAGs.

Five of the AA enzymes (*n*=7) were prevalent in the pig population (>90 subjects). AA10 was majorly found in *Enterococcus_B hirae* (52.9 %), AA2, AA3 and AA6 were found mostly represented by '*Terrisporobacter othiniensis*' (28.6%), *Methanobrevibacter_A gottschalkii* (20.1 %) and *Escherichia flexneri* (17.5 %), respectively.

Cohesin (*n*=1) and SLH (*n*=1) were found in 92 and 167 subjects, respectively, and across multiple species. Cohesin was mostly found in *Ruminococcus_C* sp000433635 (15.4 %), while *Ruminiclostridium_C* sp000435295 carried the most SLH (13.0 %).

Seven enzymes, present in between 20 and all subjects (*n*=167, comprising piglets and mothers), were found to primarily derive from a single species (>70 %). These were: CBM44 (* Clostridium* _P *perfringens*), GT44 (* Chlamydia suis *), CBM75 (* Ruminococcus* _F *champanellensis*), CBM71 (An172 sp002160515), GH44 (* Ruminococcus* _C sp000433635), GH54 (RC9 sp000432655), CBM83 (* Agathobacter* sp900317585).

In order of number of genes per genome we found: GT (median=6.3; mean=30.0), CE (median=5.5; mean=25.3), GH (median=3.7; mean=16.2), SLH (median=2.0; mean=12.7), AA (median=2.0; mean=11.2), CBM (median=2.0; mean=6.3), PL (median=1.5; mean=5.7), cohesin (median=1.0; mean=3.5). Gene counts of enzymes per GTDB species are reported in Table S1.

### Piglet carbohydrate proteome across time

**Fig. 4.**
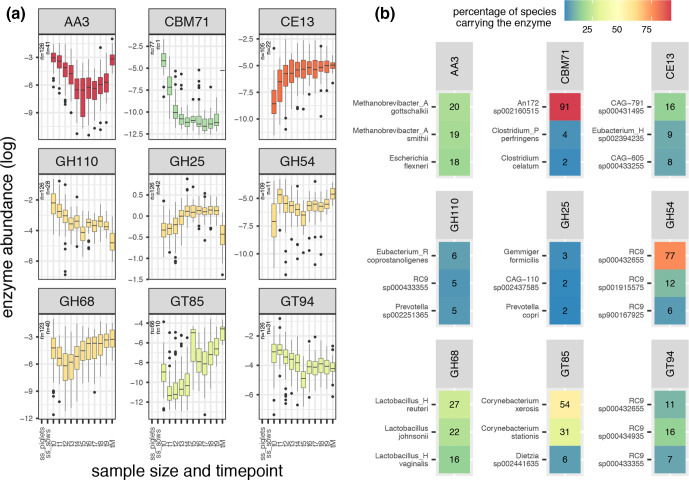
Time trend of several enzymes and their species representation. **(a**) log-transformed normalized abundance of enzymes over time is represented. Shifts in log-transformed abundance over time is visualized from time point 0 (*t*0; piglets aged 3–4 weeks) to time point 8 (*t*8; piglets aged 7–8 weeks) for several enzymes. *t*M represents the abundance of each enzyme in the mothers (single time point); the sample size for piglets and mothers carrying each enzyme is reported next to the boxplots within each panel. (**b**) Panels report the top three species carrying each enzyme in their MAGs (based on GTDB taxonomic clustering of MAGs). A significant change in abundance was recorded between different time points (Kruskal pairwise comparison with Bonferroni correction) for the enzymes shown. The full list of time trends and percentages of each species per enzyme are reported in Table S1.

Of the 294 CAZy enzymes in our dataset, 234 showed a significant change (Bonferroni adjusted *P* <0.05) (Table S1), when comparing the abundance between any time point by pairwise Wilcoxon test. The abundance shifts of these enzymes at different time points in the piglet population and their abundance in the mothers’ population are reported in Fig. 4 and Table S1.

A number of enzymes increased in abundance over time in the piglet population to reach similar abundance levels to the mothers’. Among these were: CE13, GH42, GH32, GT53, GT87, GH1, GT85, CBM25, CBM4, CE5, GT101, CBM26, GT103 and GT85. Other enzymes decreased in abundance over time in the piglet population and their abundance in the mothers’ population was as low or lower than the abundance recorded at the last time point for the piglets. Among these were: GH123, GH29, GH110, GH33, GH30, GH109, GH2, GH35, CBM35 and CBM9. A number of enzymes followed an upwards or downwards trend with time in the piglet population while their abundance in the mothers population was lower or higher, respectively, than the abundance recorded at the last time point in the piglet population. Among these were: GT66, CBM51, GH25, GH37, CBM13, CBM67, GH101, CBM44, GT46, GT22 and CBM6.

## Discussion

Previous efforts have applied metagenomics to survey the diversity of pig gut microbiota [83], but have not focused on the post-weaning microbial community nor have they used an approach that generates MAGs. Bowers *et al.* (in 2017) described genomes with a completeness of at least 90 % and a contamination rate lower than 5 % as nearly complete genomes [84]. In this study, we generated over 50 000 MAGs from a dataset of 27 billion read pairs, of which 12 486 MAGs are predicted to represent nearly complete genomes of bacterial or archaeal organisms. While we previously reported the results obtained from an assembly-free analysis of community structure [21], here we report the composition and predicted carbohydrate proteome of the microbial community using MAGs constructed from the dataset. Using the MAGs and their abundance across the samples, we were able to track compositional and functional changes over a 5 week period in 126 post-weaning piglets.

As previously observed [21], we confirm the large time factor driving the compositional shift of post-weaning piglet gut microbiota samples. The temporal shift appears regardless of the approach-dependent grouping of MAGs (CheckM taxonomic clustering, 99 % ANI clustering or GTDB taxonomic clustering), or of the filtering criteria (completeness and contamination, length filtering, no filtering, respectively) and, hence, the resulting number of MAGs taking part in the analysis (12.4×10^3^, 22.4×10^3^, 51.2×10^3^, respectively). We also applied a number of normalization methods in parallel, among which we chose median sequencing depth, rarefaction and proportions. The temporal shift was evident when applying any of these normalization methods, although it must be noted that the use of proportions to normalize library size can lead to a higher discovery rate of false correlations (i.e. spurious correlations of ratios) [85–89]. Bias can also arise from the nature of organisms in the sample as a consequence of distinct genomic G+C contents [90], causing preferential affinity of the polymerase, thereby influencing the representation of species in the sample.

Factors such as host weight, breed and small differences in age correlate with microbial composition, as we found by our previous analysis with unassembled metagenomic reads [21] and in this study with the use of MAGs. To assess the correlation of microbial composition and small age differences among the hosts, the dataset was reduced to include only piglets of the same breed, thereby avoiding breed as a confounding factor. The temporal signal in our dataset was in fact sufficiently strong in the first 2 weeks of the trial that piglets differing by just 3 days of age separated cleanly in the first principal component, and a number of GTDB-assigned species were found to be associated with these small age differences.

Highly structured compositional changes over time were detected. Possible factors that underlie these compositional changes include the exposure to a new environment, the transition from breast-milk to solid food [91–93], which took place upon the start of the trial, and a higher tolerance for new species immediately after weaning [94–96]. Supporting the latter, it is known that during weaning, the immune system undergoes training to recognize pathogens by initially tolerating a large number of species, so as to develop the necessary antigens upon encounter [97, 98]. Thompson *et al.* [96] report that the microbiota of piglets of 3 weeks of age is particularly dynamic and environmental factors start to be seen at this stage [96]. In fact, over 80 % of the GTDB clustered MAGs that showed significant abundance shifts during the first week of post-weaning were showing positive fc. This is also reflected by the major increase in microbial richness we measured by community composition analysis of the GTDB clustered MAGs, as well as by phylogenetic diversity analysis of the metagenomic reads in our previous study [21]. Nearly 40 % of the species that increased during the first week of the trial (*t*0–*t*2) continued to increase during the following week (*t*2–*t*4), reflecting the fact that these species kept taking up a larger portion of the microbial community. This is confirmed by the increase of evenness (as measured by balance-weighted phylogenetic diversity) we recorded for this time interval in our previous study [21]. The third week of the trial was the last week in which the majority of the taxonomic shifts were positive fc (>80 %; piglets aged between 6 and 7 weeks), after which species richness stabilized. In summary, in this study, the first 3 weeks (piglets aged between 3 and 7 weeks) represented the establishment of a new microbial community where a large number of species bloomed.

After a brief transition period (fourth week; piglets aged between 6 and 8 weeks) during which only a few species are lost and acquired, a microbial community consolidation phase starts, where species start to die off, and a smaller number of dominant taxa remain, reflective of an adult gut [99]. This statement is supported by Thompson *et al*. [96], who determined that at 6 weeks of age CD8^+^ T cells infiltrate the intestinal tissue and the mucosal lining resembles that of an adult pig [96]. In the human gut, *Prevotella* are known to be acquired post-weaning as a consequence of a substrate shift from breast-milk to solid food in infants [91]. Notably, in this study, 16 *Prevotella* species established during the first and the second week (piglets aged between 3 and 6), 12 of which were found to significantly drop in abundance during the last time interval. Previously associated with human milk consumption in infants [91], *Bifidobacterium* species dropped significantly in abundance during the last 2 weeks, between the third and the fourth week post-weaning, suggesting this shift to be due to the diet change from breast-milk to solid food.

Studies have reported the health promoting effects of *Lactobacillus amylovorus* [100, 101], its survival through gastric pH, its ability to adhere to cells and the demonstrated efficacy against the growth of certain pathogens [100, 101]. In this study, *Lactobacillus amylovorus* follows a specific trend, by gradually increasing in its abundance from piglets aged 3–4 to 5–6 weeks, to then gradually decrease in the hosts, suggesting that the administration of probiotics containing this species, to 6-week-old piglets, may not lead to a successful colonization.

Mapping of predicted ORFs generated hits with a high sequence similarity against the CAZy database, spread across eight enzyme classes and containing 294 unique enzymes. Similarly to the rumen metagenome [102], half of the enzymes were of the GH class and over a third were of the GT class, the first known to break down sucrose, lactose and starch [103], the latter known to assemble complex carbohydrates from activated sugar donors [104]. The proportions of the most abundant enzymatic classes in this study (50 % GH; 35 % GT; 12 % CE; 1 % PL) roughly matched the proportions suggested by El Kaoutari *et al.* [103] (57  % GH; 35 % GT; 6 % CE; 2 % PL), who generated a profile of the human carbohydrate repertoire by mapping 177 reference human gut microbial genomes against the CAZy database [103]. We reported the representation of these CAZymes within each specific taxonomic group at lower (e.g. species) and higher levels (e.g*.* phylum) based on GTDB taxonomic clustering, and we reported on the carbohydrate enzymes abundance shifts over time in the post-weaning piglet.

A number of enzymes were found to change gradually in abundance over time, to reach levels that are similar to those found in the piglets’ mothers. As it could be expected due to the large difference in age between the piglets at the last sampling point and the mothers, the time trend for a minority of the total enzymes changed gradually in the piglets, but did not reach the lower or higher level measured in the mothers. A number of enzymes known to break down animal glycans that are present in milk (GH2, GH20, GH92, GH29, GH95, GH38, GH88) [103] decreased with time in the piglet population, while, among others, enzymes reported to break down peptidoglycans (GH25, GH73), starch and glycogen (GH13), and sucrose and fructose (GH32) [103] followed an upward trend.

The data we report in this study address a knowledge gap in the association of carbohydrate active enzymes to microbial species. Similarly to the rumen metagenomic study [102], we found a larger proportion of GT enzymes in the phylum *Euryarchaeota *, and a larger proportion of CBM and PL enzymes in the phylum *Fibrobacterota *. However, it must be noted that the method of annotating CAZymes by mapping genomes against a reference database, as for many other reference-based analyses in microbial metagenomics, may suffer bias due to the limited representation of diversity in the database. In the case of CAZymes, the approach may accurately represent the CAZyme profile of better-represented genomes or better-studied enzyme families, while underestimating the CAZyme profile of genomes for which reference genomes are lacking.

We found enzymes to be either (i) common to many species and to higher level taxonomic assignments, indicating functional redundancy; or (ii) shared among species within the same genus; or (iii) specific to single species. Common enzymes restricted to closely related species were: AA10, found predominantly in the genus *Enterococcus *, CBM40 in *Clostridium *, PL31 in *Ruminococcus *, CE5, GT53, GT85 and GT87 in *Corynebacterium *, GT21 in *Desulfovibrio *, GT66 and GT81 in *Methanobrevibacter *, GT6 in *Gemmiger *, GH68 in *Lactobacillus *, PL21, GH54 and GH55 in RC9 genera. Members of the same genus also significantly differed in their enzymatic repertoire, both in the total number of genes encoding enzymes per genome, as well as in the number of different enzymes a genome bears, showing that closely related organisms have evolved to display large differences in behaviour [9, 105]. This was particularly evident among species of the genus *Prevotella *, potentially suggesting a high degree of specialization in preferred food sources among closely related species. The synthesis of knowledge of enzyme function and substrate-based activity coupled with knowledge of the species carrying these enzymes in their genome can be of high value to the design of probiotic and prebiotic formulations in the livestock setting (e.g. methane emission control; improvement of substrate energy efficiency), as well as in the human setting (e.g. diet).

## Supplementary Data

Supplementary material 1

Supplementary material 2
