# Supplementary material for: Post-weaning shifts in microbiome composition and metabolism revealed by over 25 000 pig gut metagenome-assembled genomes
Source: Microb Genom. 2021 Aug 9;7(8):000501. doi: 10.1099/mgen.0.000501 (PMC8549361; doi:10.1099/mgen.0.000501)
Supplement: Supplementary material 1 [file mgen-7-0501-s001.pdf]

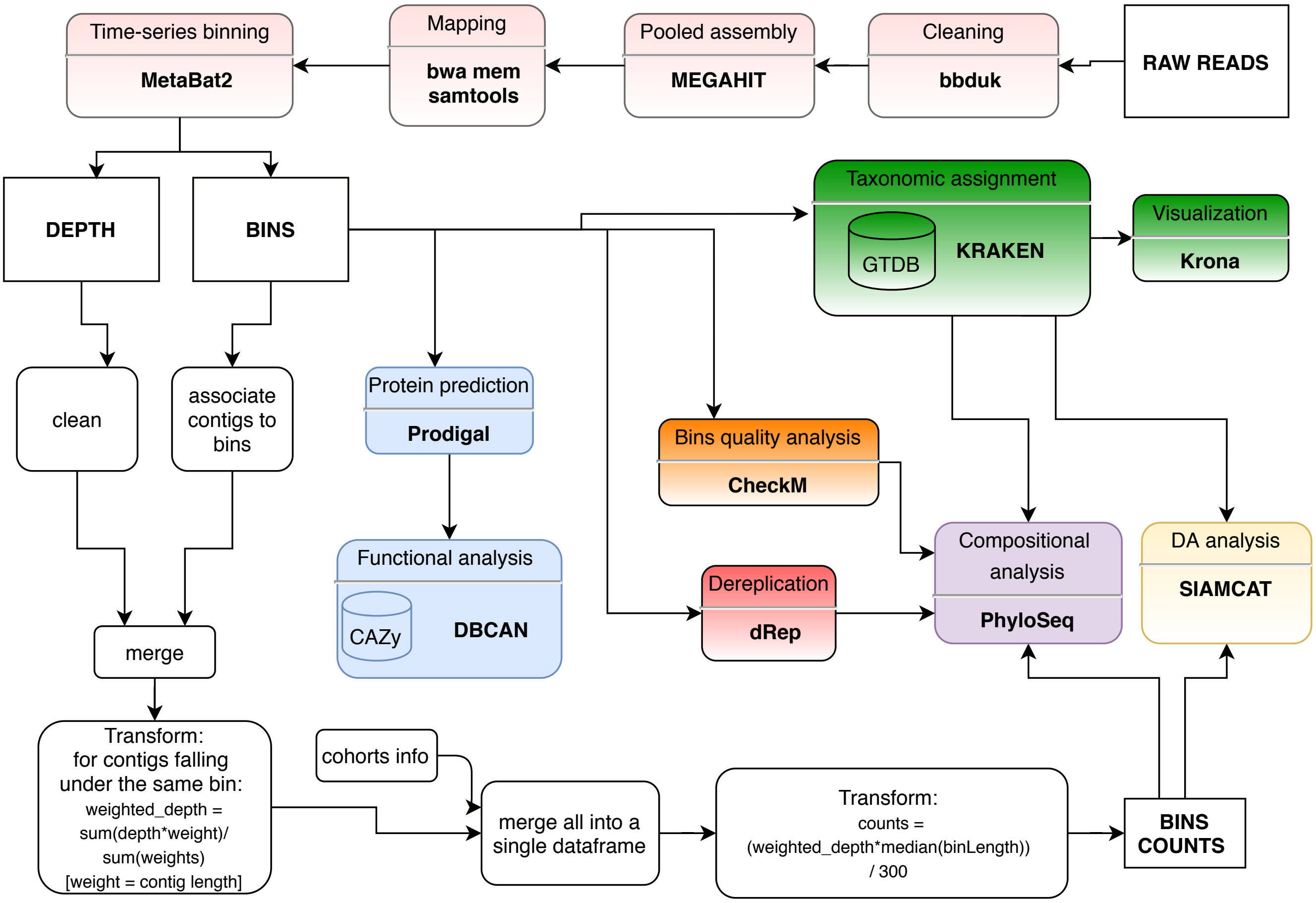

Supplementary Figure 1. Workflow of sequence data processing and analysis.

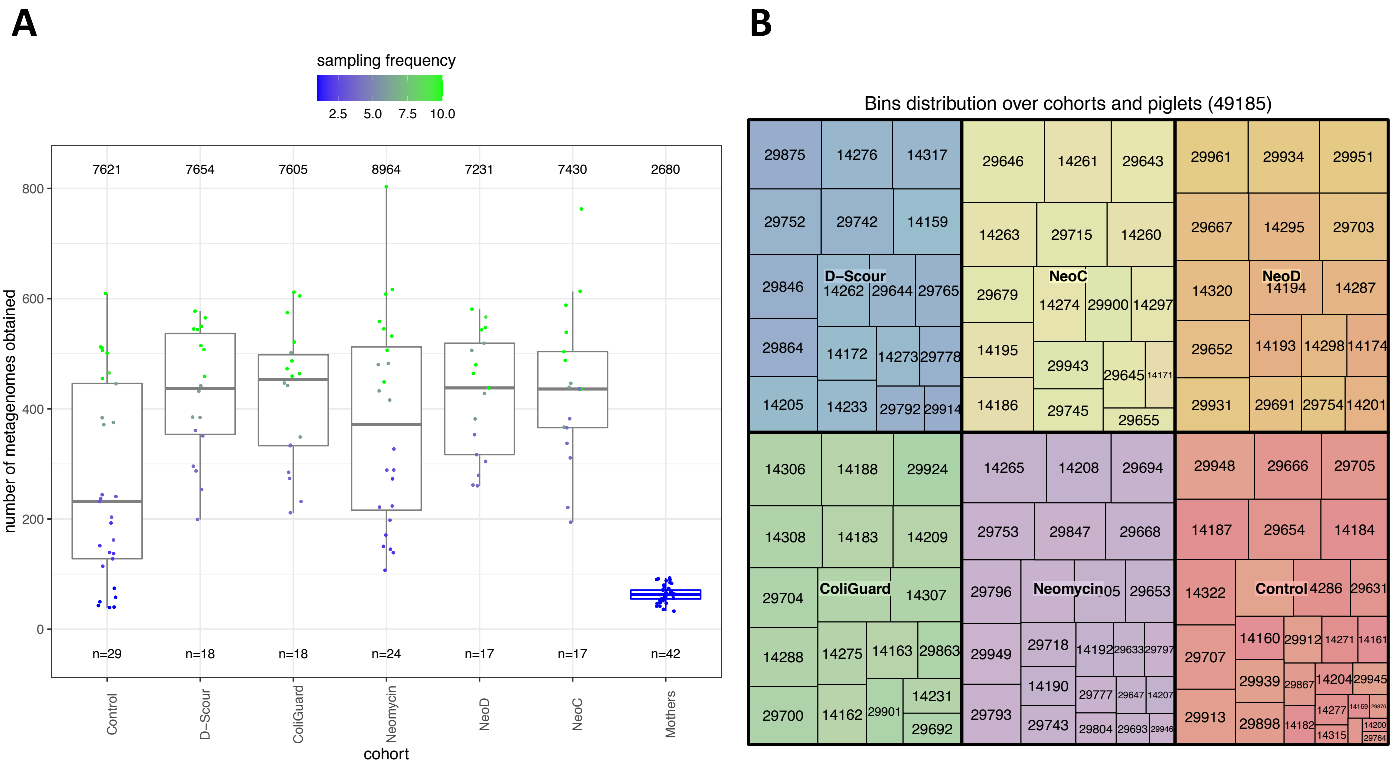

Supplementary Figure 2. Distribution of bins over subjects and cohorts.

A) In total, mothers have been sampled once, while piglets have been sampled between 1 and 10 times (median: 6.0; mean: 6.18) over a period of 5 weeks. The control and neomycin cohorts, in which 12 and 6 subjects were euthanized during the first week of the study, had a mean of 4.5 and 5.6 time point samples and a mean bin count of 262.8 and 373.5 bins per subject, respectively. In the other cohorts, with a mean of 6.0 time point samples per subject, an average of 430.3 bins per subject was obtained. **B)** The higher number of time point samples available from the subset group is reflected in the higher number of MAGs obtained from these piglets.

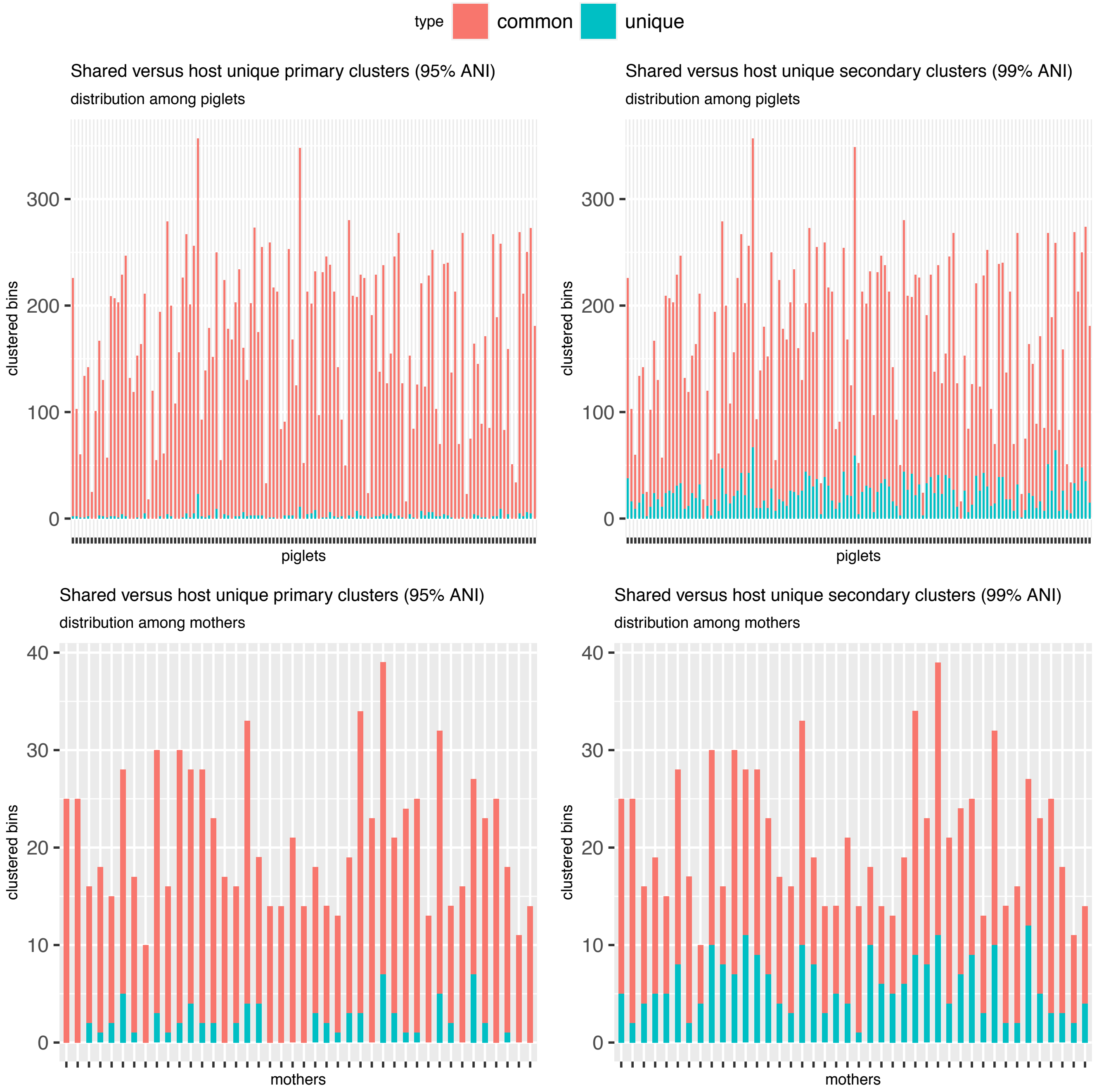

Supplementary Figure 3. The fraction of MAGs that are unique to individual subjects when clustered at 95% and 99% ANI.

Primary clusters (95% ANI) (**A-B**) were shared across subjects at a higher rate than secondary clusters (99% ANI) (**B-C**): 98.6% and 86.2% among piglets (**A-C**) and 91.4% and 72.1% among the mothers (**B-D**), respectively, delineating strain specificity. It appears that each individual subject harbors a specific set of ANI clustered MAGs (95% ANI or 99% ANI) that are unique to the subject, as well as a set ANI clustered MAGs (95% ANI or 99% ANI) that are common among subjects. A larger fraction of unique ANI clustered MAGs (either at 95% or 99% ANI) is seen among the mothers than among the piglets.

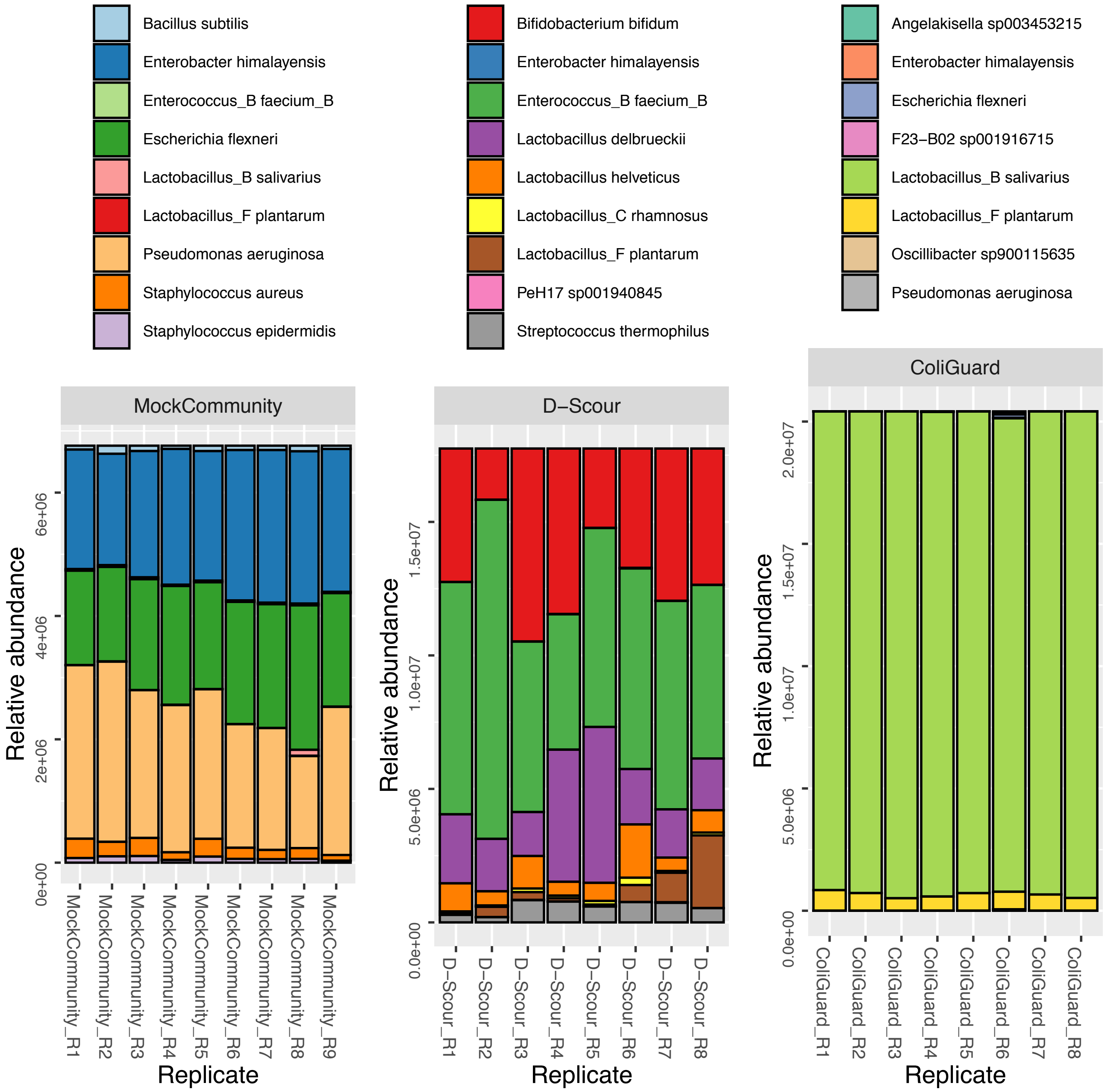

Supplementary Figure 4. Taxonomic profile of the positive controls.

Taxonomic profile is obtained from PhyloSeq analysis of GTDB clustered MAGs of the positive controls samples. The taxonomic profile within each replicate is displayed in relative abundance. Each replicate sample is normalized by median sequencing depth.

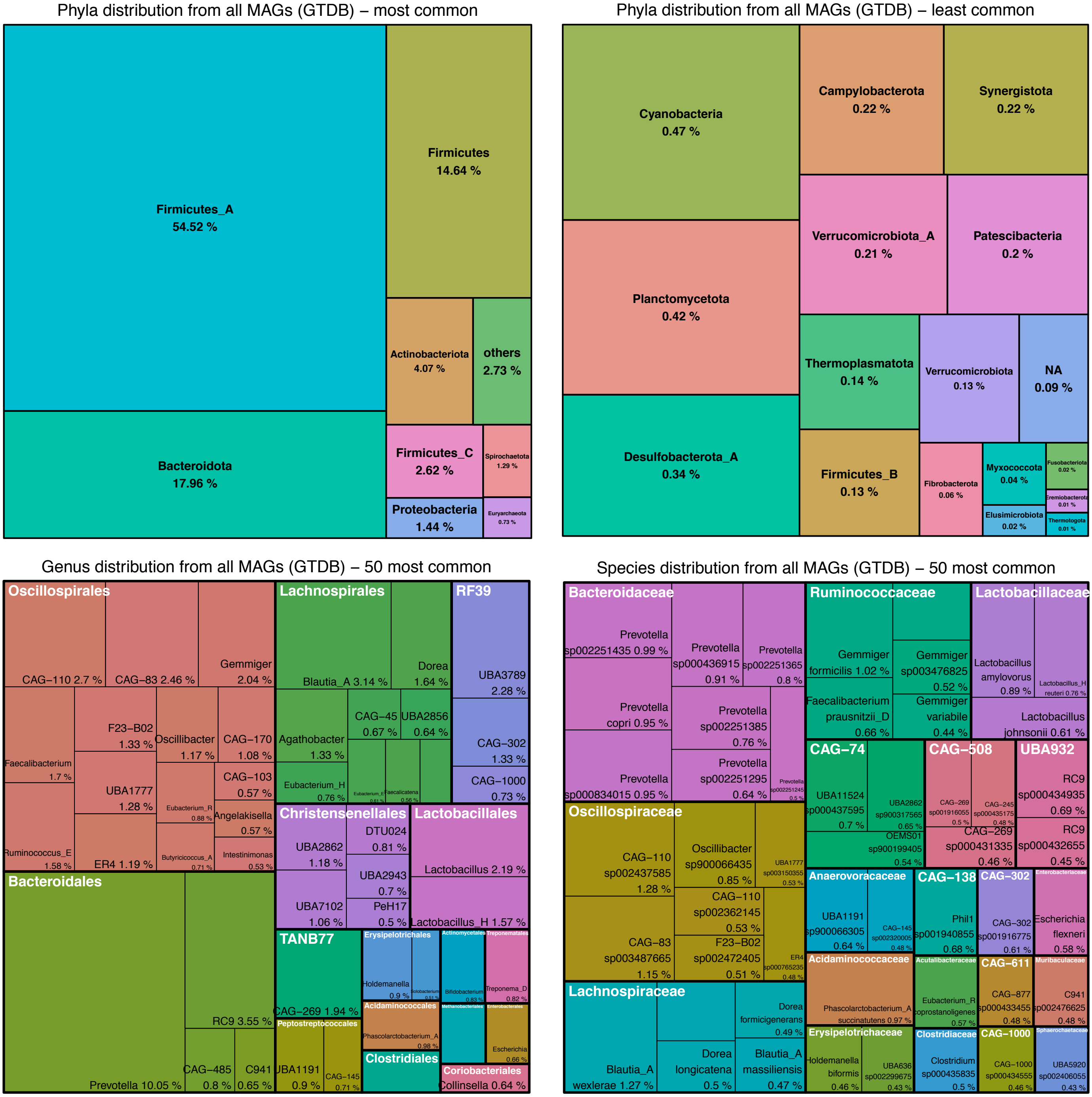

Supplementary Figure 5. Taxonomic composition of the piglet gut.

Tree maps were built from MAGs taxonomically clustered with GTDB, without scaling for the relative abundance of each MAG. This accounts for prevalence of the MAG in the pig population (*e.g.* if the same taxon occurs in multiple piglets it can get counted more than once). The most common Phyla (top left), the least common Phyla (top right), the 50 most common Genera colored by Order (bottom left), and the 50 most common Species colored by Family (bottom right) are shown.

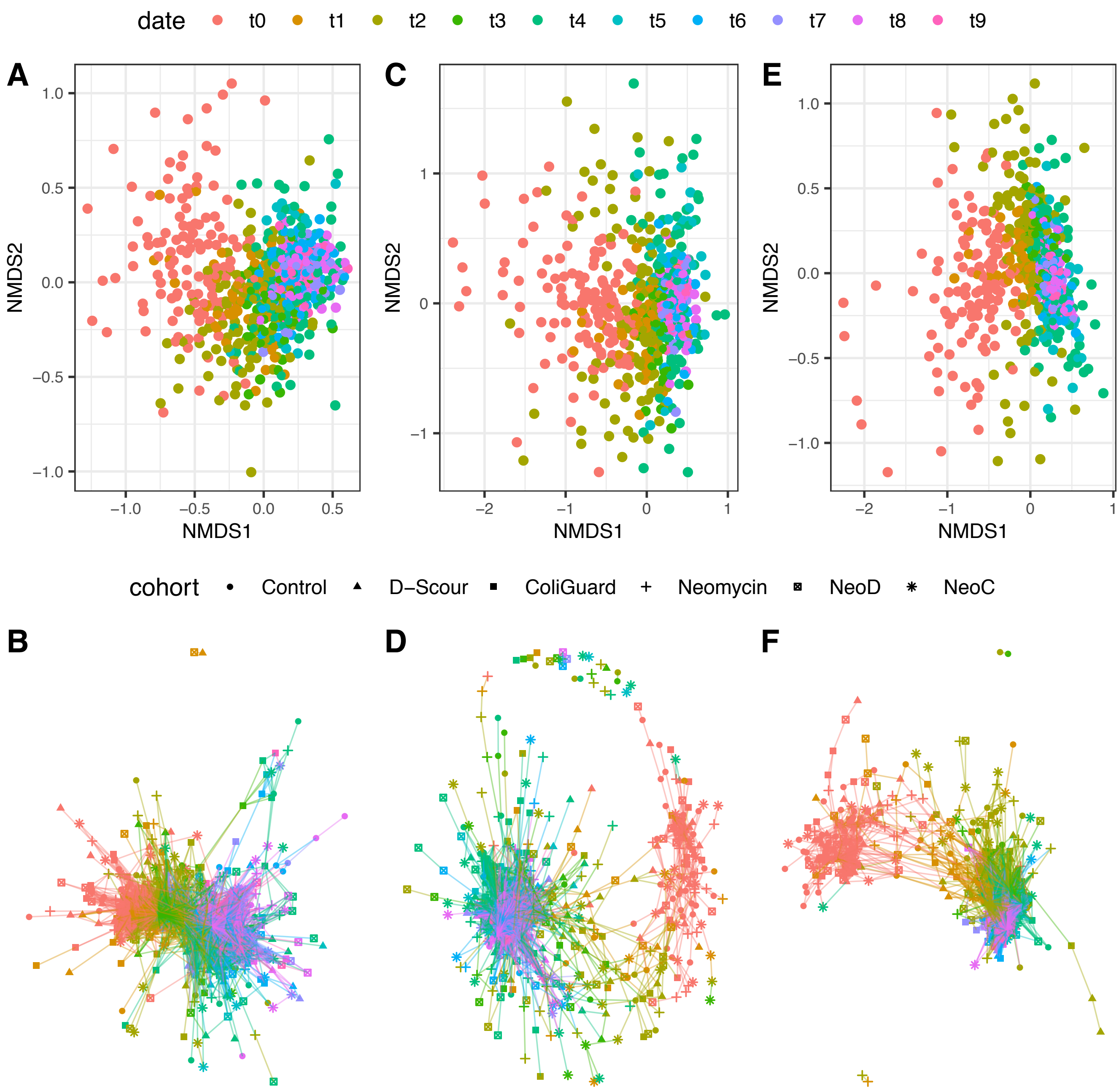

Supplementary Figure 6. Temporal shift of MAGs from PhyloSeq analysis.

NMDS analysis (top) and network analysis (bottom) with PhyloSeq from nearly complete- CheckM clustered MAGs (12.4k) (**A-B**), 99% ANI clustered MAGs (22.4k) (**C-D**), and GTDB clustered MAGs (51.2k) (**E-F**).

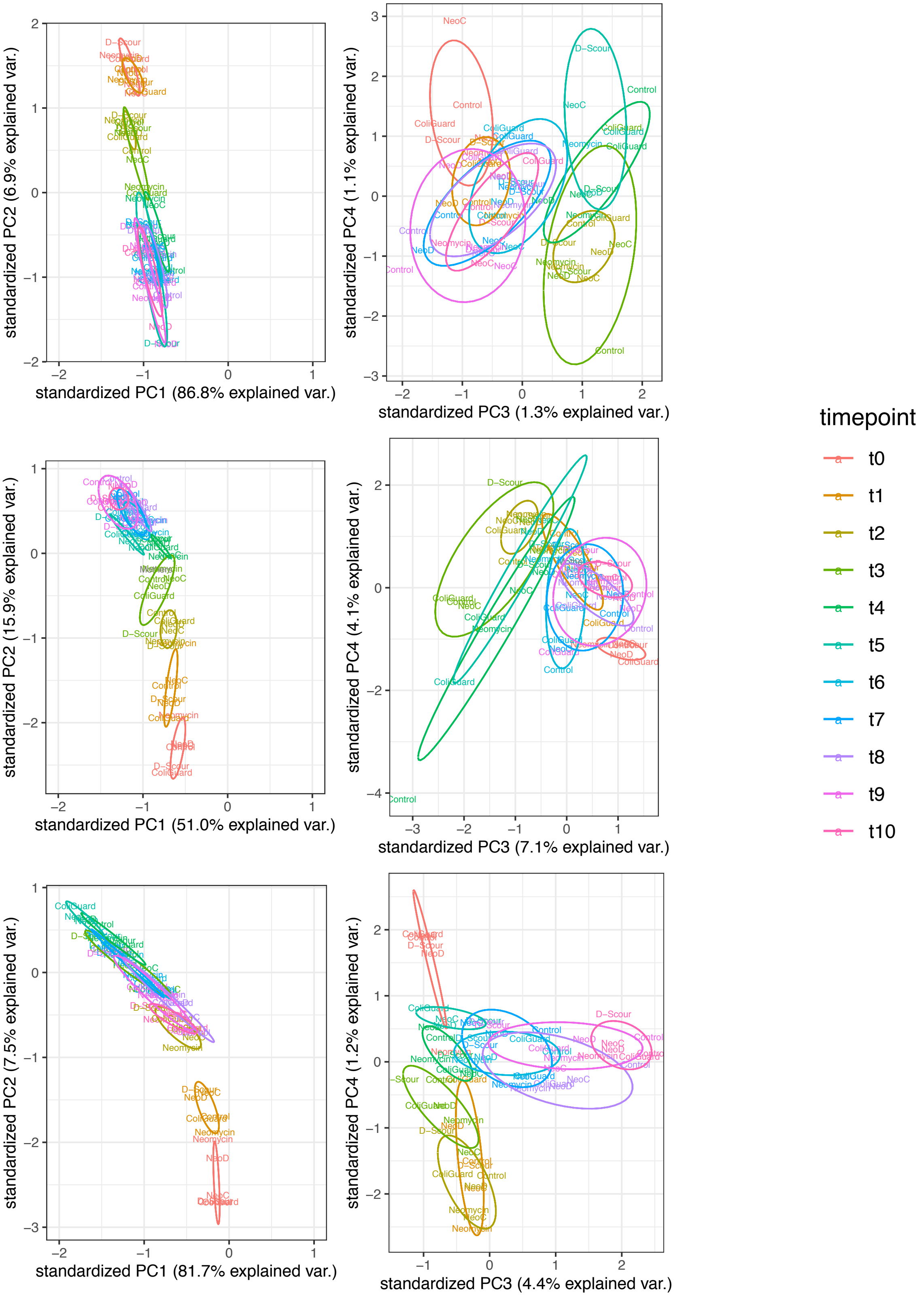

Supplementary Figure 7. Temporal shift of samples from CheckM clustered MAGs, dRep- ANI clustered MAGs, and GTDB clustered MAGs.

Principal component analysis from nearly complete CheckM taxonomically clustered MAGs (12.4k) (top), ANI clustered MAGs (22.4k) (middle), and GTDB taxonomically clustered MAGs (51.2k) (bottom), across the first and the second (left), and the third and fourth principal components (right). Prior to PCA, the data was normalized by proportions, the average proportion was computed by time point and treatment cohort, and the centered log-ratio transformation was applied. Labels report the treatment cohorts: Control, D-Scour, ColiGuard, Neomycin, NeoD (Neomycin followed by D-Scour), NeoC (Neomycin followed by ColiGuard).

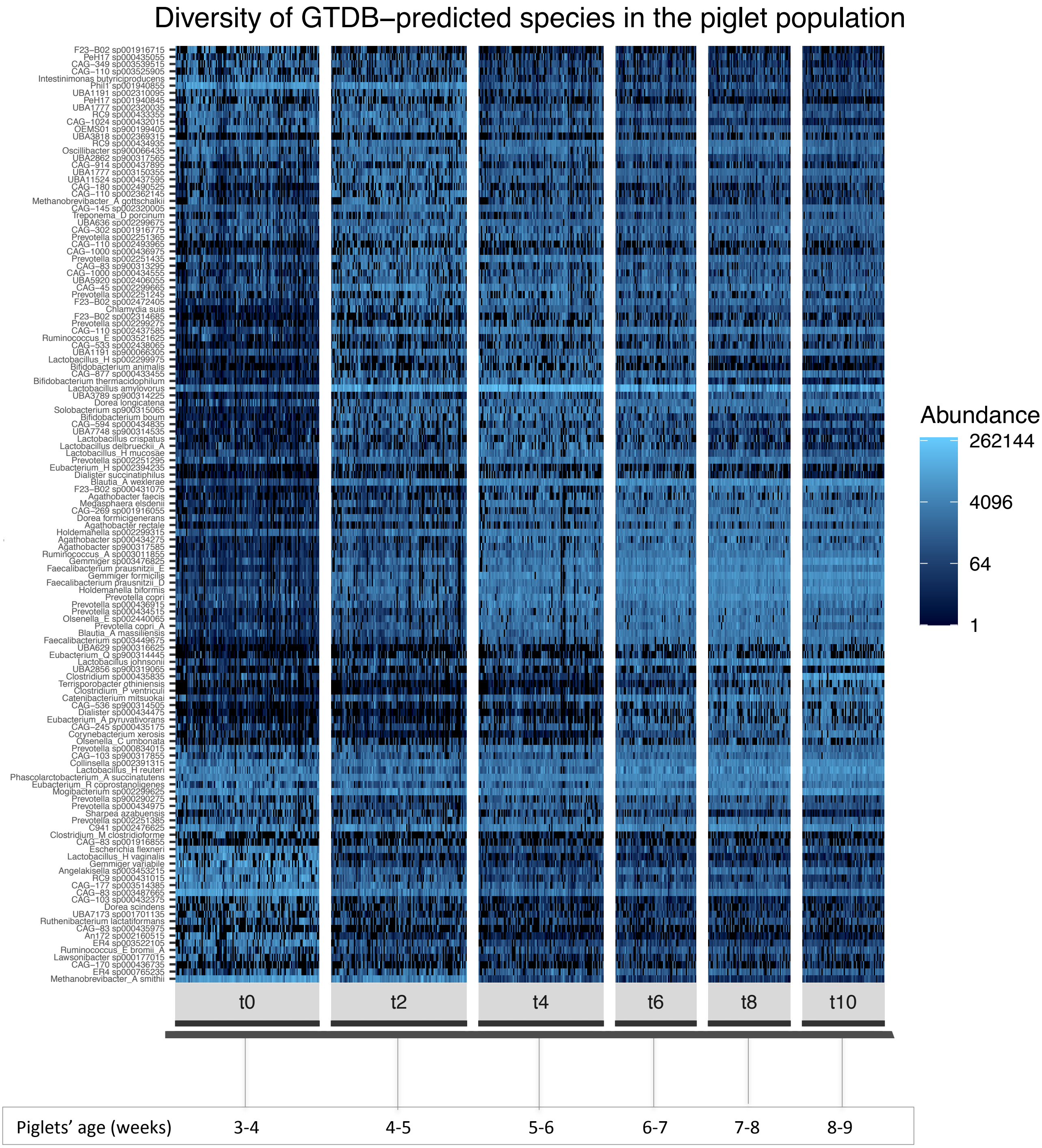

Supplementary Figure 8. Microbial species abundance profile of the piglet gut over time.

Panels represent time points from t0 (piglets aged 3 to 4 weeks old) to t10 (piglets aged 8 to 9 weeks old). Samples were pruned (exclusion of samples with ca. <10,000 read counts) and normalized by rarefaction. Prior to plotting, taxa were filtered to include the most abundant taxa present in at least 20% of the piglet samples. Of these, the hundred thirty taxa with the highest variance among samples were plotted. Analysis is performed with PhyloSeq.

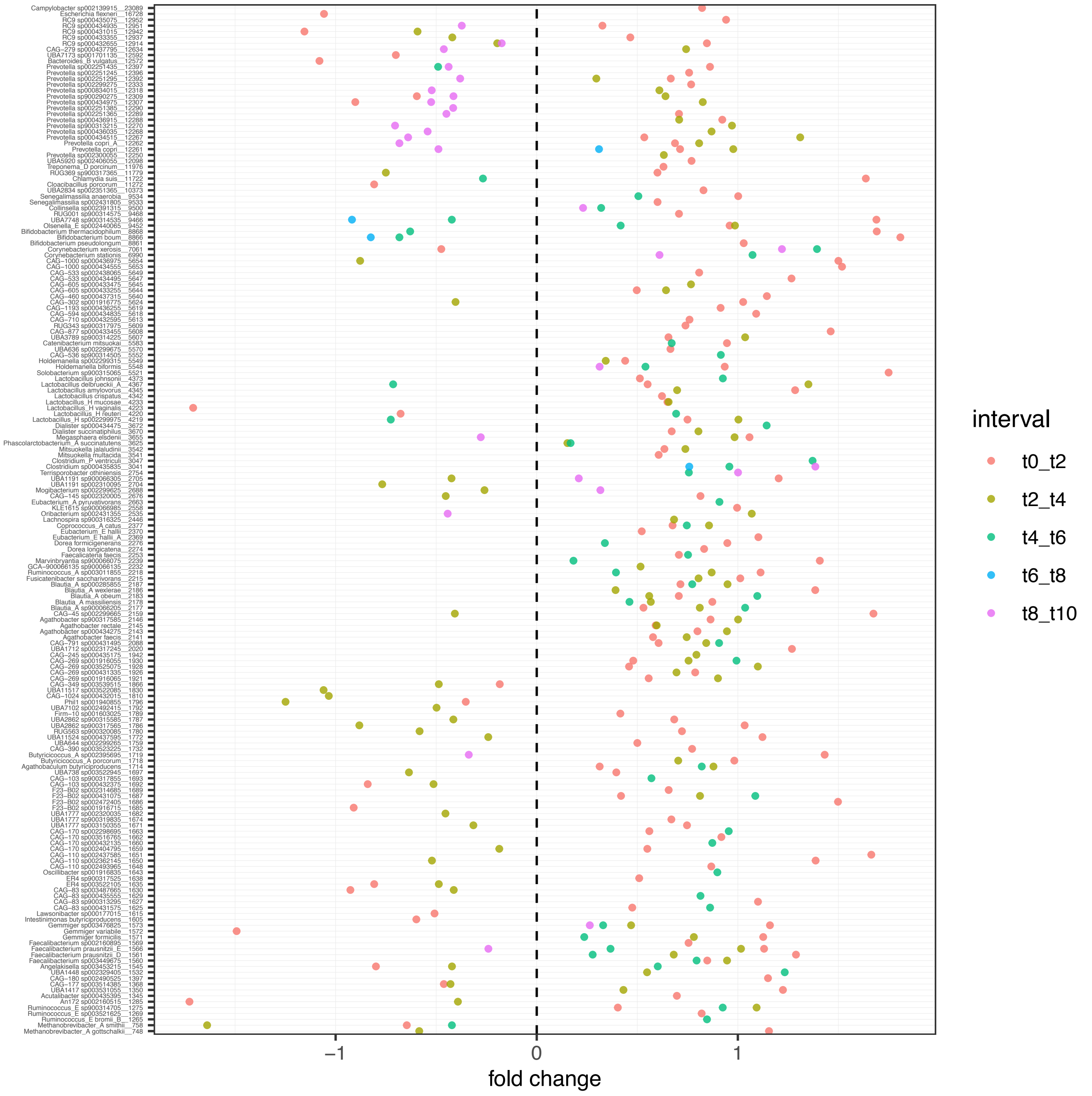

Supplementary Figure 9. Species significantly shifting in abundance at all time intervals.

Significant shifts (FDR adjusted *p* value < 0.05) between time points during the trial are displayed for all GTDB clustered MAGs. A large number of species increased in abundance during the first week (red dots). The following week (yellow dots) several kept increasing the (fc > 0; top and middle right), wheras some descreased (fc < 0; near bottom left). Wheras nearly all *Prevotella* increased during the first and second week (red and yellow dots, respectively), nearly all decreased during the last time interval (purple dots; fc < 0; top left). Species are sorted by phylogenetic tree node number.

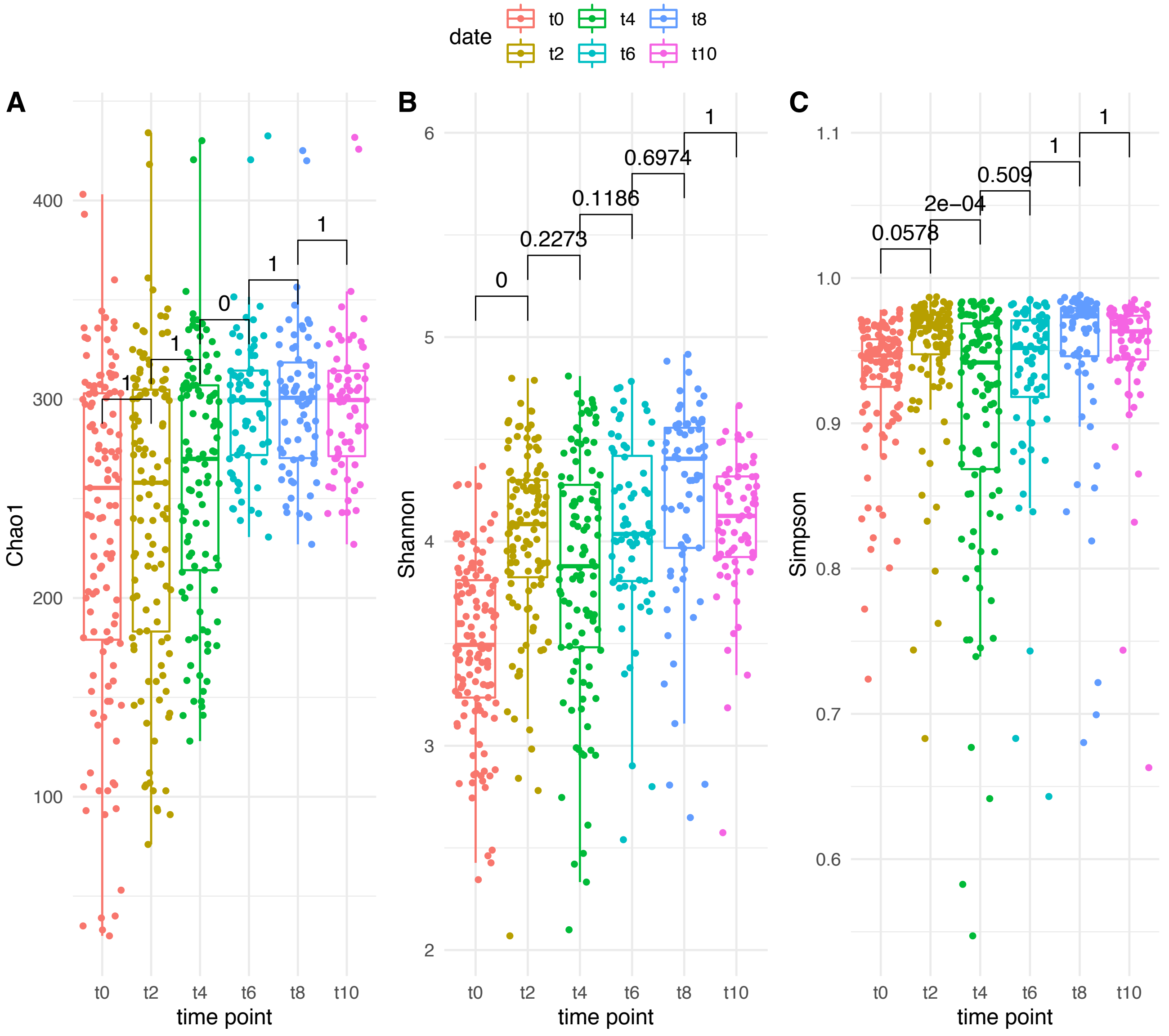

Supplementary Figure 10. Sample diversity across time.

Samples were rarefied and diversity was obtained with PhyloSeq. Diversity measures displayed **A**) Chao1: abundance-based estimator of species richness; **B**) Shannon: estimator of species richness and species evenness, with more weight on species richness; **C**) Simpson: estimator of species richness and species evenness, with more weight on species evenness. Sample diversity scores from different time points were compared by t-test and significance values were adjusted with the Bonferroni method. GTDB taxonomic clustering of MAGs was used.

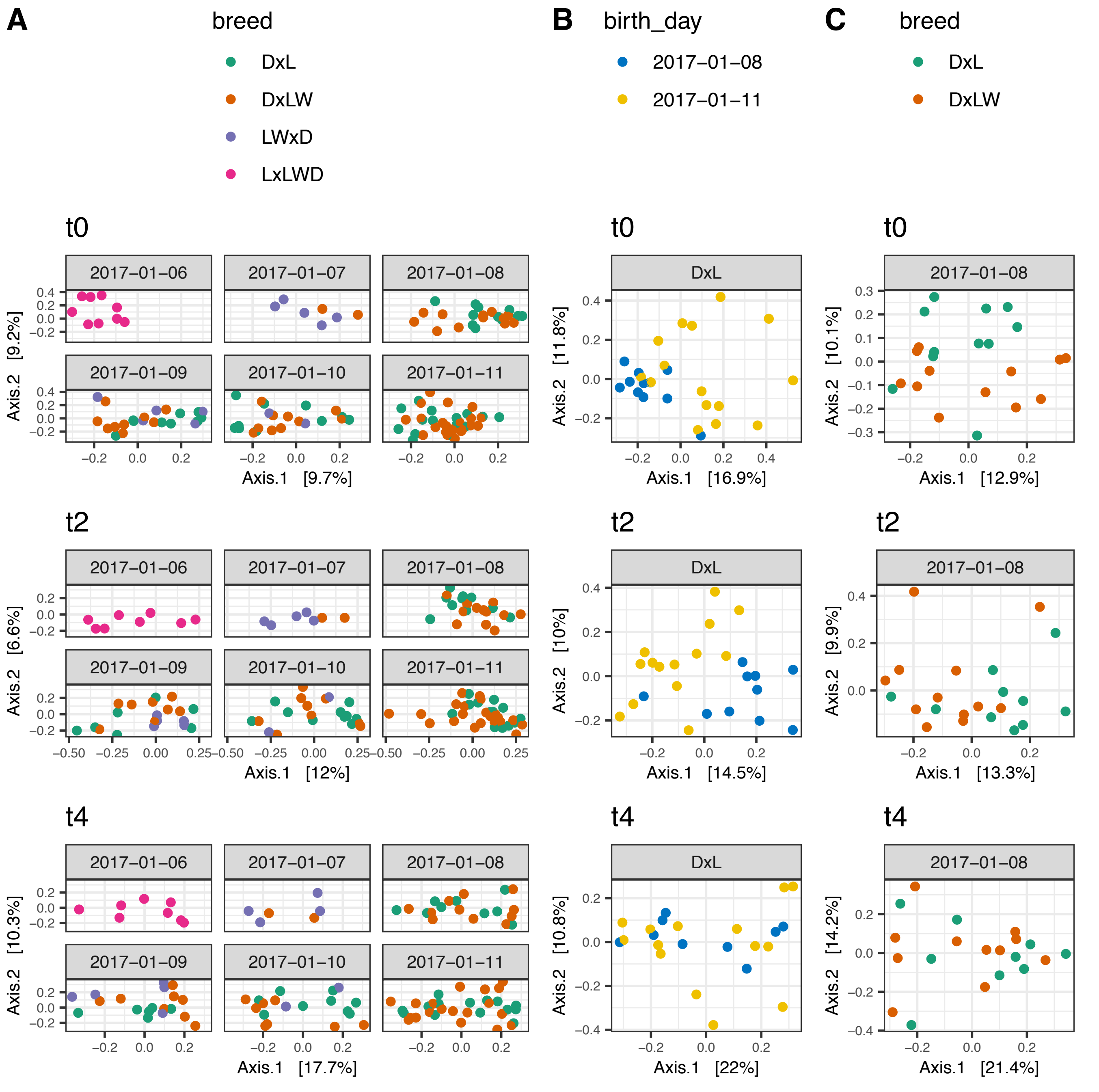

Supplementary Figure 11. Age and breed correlations with composition.

A principal component analysis was performed for all samples (**A**), for a subset including one breed and two age groups (**B**), and for a subset including one age group and two breeds (**C**). Metadata with breed and age was joined to the principal components coordinates generated with Phyloseq. GTDB taxonomic clustering of MAGs was used. Each plot shows the principal components 1 and 2, reporting the percentage of variation explained. Significance of the correlations was determined with the Dunn test and *p*-values were corrected with the Bonferroni method. We refer to Supplementary File 1 for all the generated *p*-values.

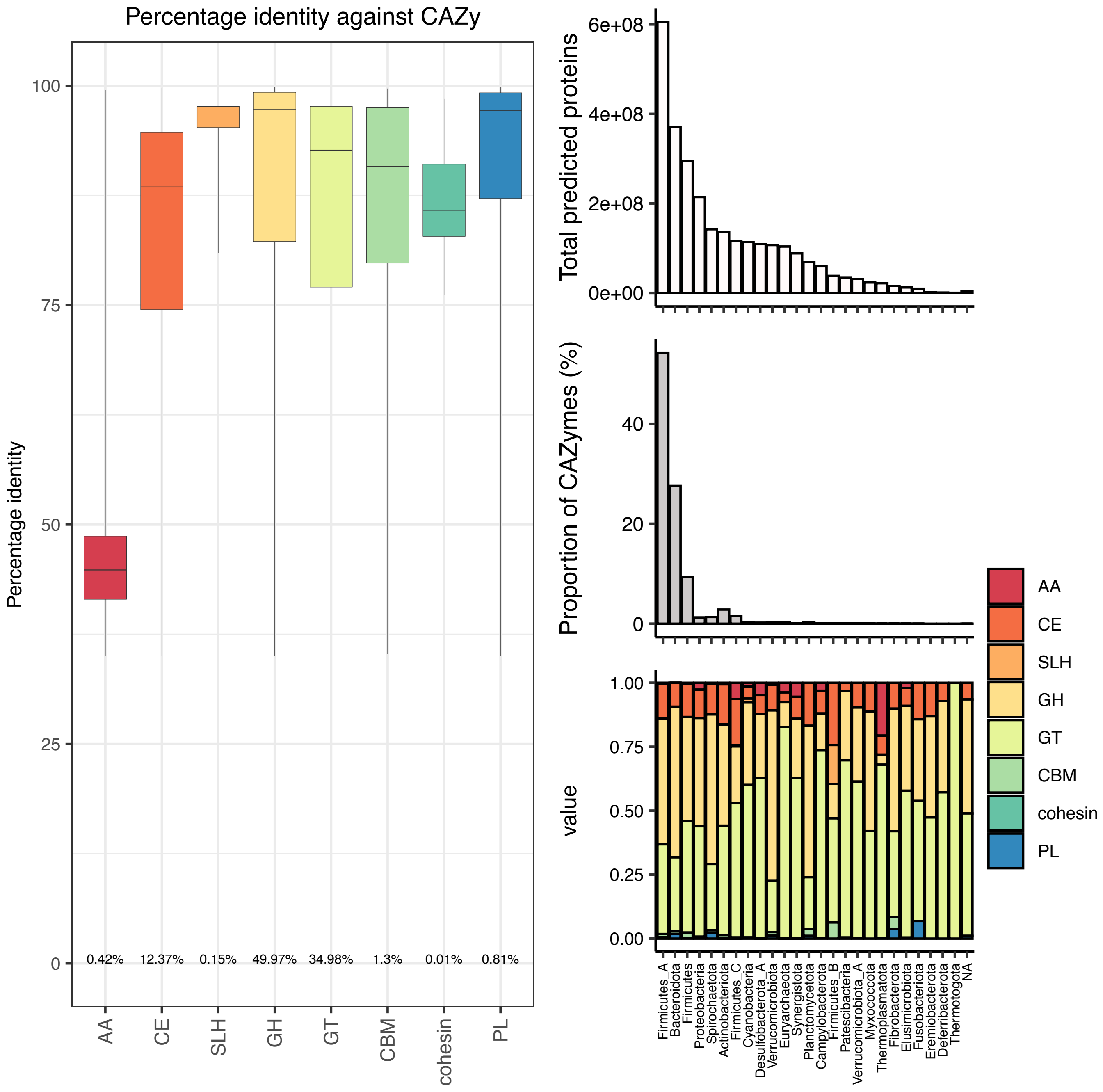

Supplementary Figure 12. Sequence identity against the CAZy database and proportions across Phyla.

Sequence identity of all predicted proteins against the CAZy database is shown (left). Numbers on the bottom of the boxplots report the proportion each enzyme class that is present in our dataset. Distribution of predicted proteins (top right), distribution of CAZy enzymes (middle right) and proportions of enzymatic classes (bottom right) across phyla are shown. GTDB taxonomic clustering of MAGs was used.

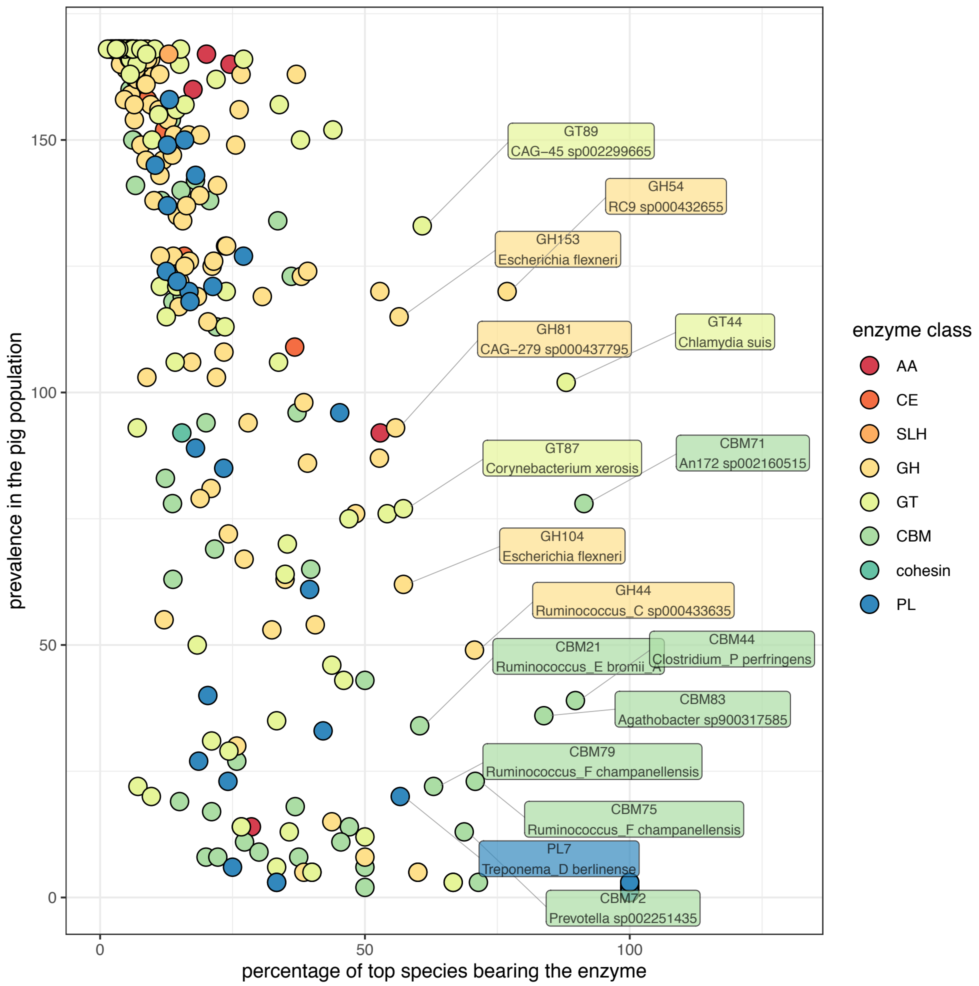

Supplementary Figure 13. CAZy enzymes in the pig population and their species representation.

Two-hundred ninety-four carbohydrate active enzymes are represented. The position along the x-axis indicates the relative abundance (as a percentage) of the species with the highest copy number of genes encoding a specific enzyme, while the y-axis represents the prevalence among the pig population (*n*=126 piglets; *n*=42 mothers). Taxonomic assignments of MAGs are based on the GTDB.

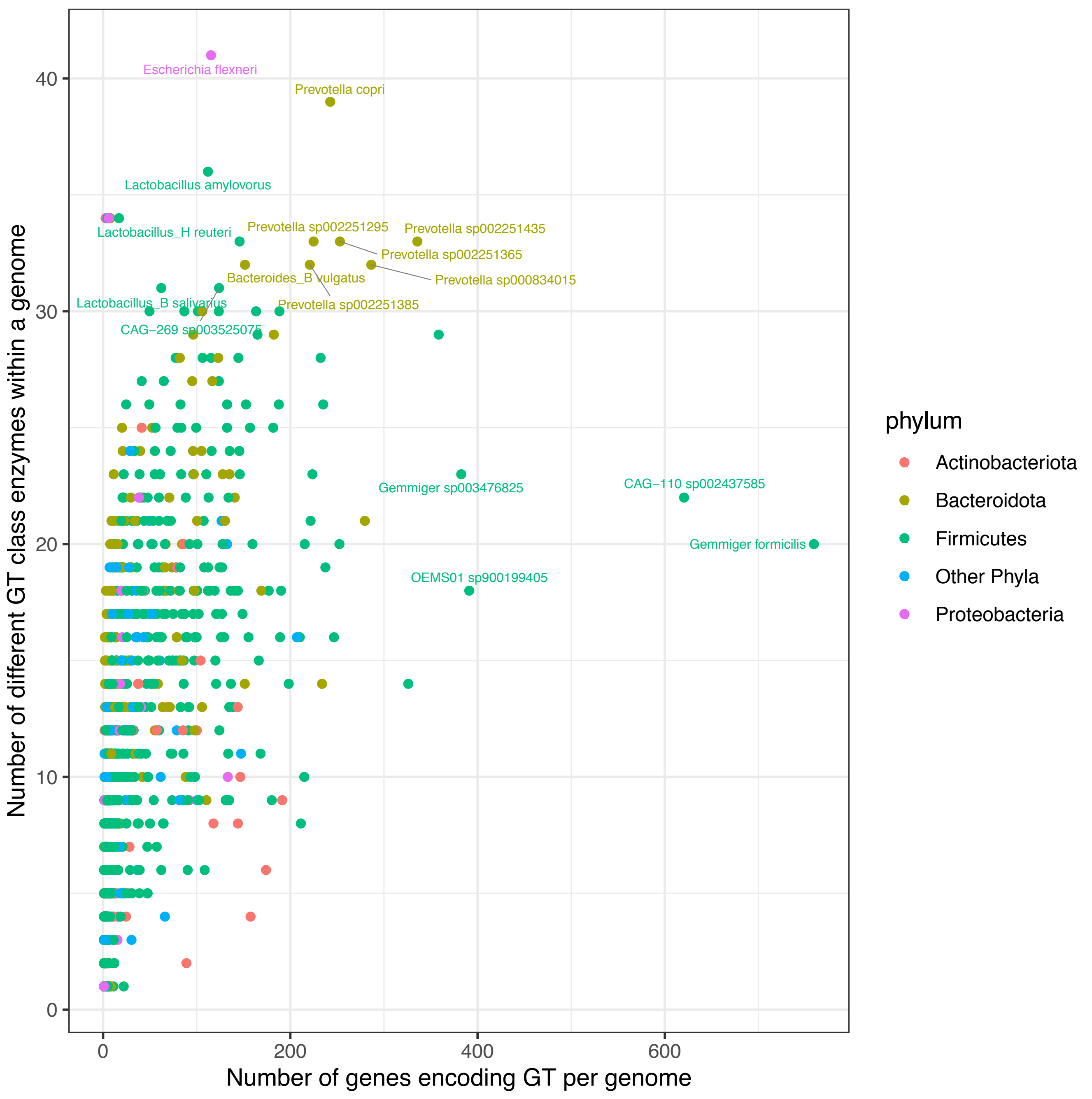

Supplementary Figure 14. Diversity of glycoside transferases.

The plot shows the number of genes encoding glycoside transferases (GTs) across species of GTDB clustered MAGs (x-axis) and the number of distinct enzymes within the GT class represented in those species (y-axis). Species of *Gemmiger* show a particularly large number of genes encoding GTs. *Escherichia flexneri* has the highest number of distinct GT enzymes among all species. A cluster of *Prevotella* species display a high count of GT genes (between 100 and 400) and a relatively high number of distinct GT enzymes. Enzymes of class GT are known to assemble complex carbohydrates from activated sugar donors (Lairso *et al*, 2008).
